# Insights into aphid prey consumption by ladybirds: Optimising field sampling methods and primer design for high throughput sequencing

**DOI:** 10.1371/journal.pone.0235054

**Published:** 2020-07-01

**Authors:** Lolita Ammann, Rosemary Moorhouse-Gann, Jordan Cuff, Colette Bertrand, Laia Mestre, Nicolás Pérez Hidalgo, Amy Ellison, Felix Herzog, Martin H. Entling, Matthias Albrecht, William O. C. Symondson

**Affiliations:** 1 Agroecology and Environment, Agroscope, Zurich, Switzerland; 2 Cardiff School of Biosciences, Cardiff University, Cardiff, United Kingdom; 3 Durrell Wildlife Conservation Trust, Trinity, Jersey, Channel Islands, United Kingdom; 4 UMR 1402 ECOSYS, INRA, AgroParisTech, Université Paris-Saclay, Versailles, France; 5 iES Landau, Institute for Environmental Sciences, University of Koblenz-Landau, Landau, Germany; 6 Instituto de Biologia Integrativa de Sistemas (Centro Mixto Universidad de Valencia-CSIC) Valencia, Spain; 7 School of Natural Sciences, Bangor University, Bangor, United Kingdom; UPMC, FRANCE

## Abstract

Elucidating the diets of insect predators is important in basic and applied ecology, such as for improving the effectiveness of conservation biological control measures to promote natural enemies of crop pests. Here, we investigated the aphid diet of two common aphid predators in Central European agroecosystems, the native *Coccinella septempunctata* (Linnaeus) and the invasive *Harmonia axyridis* (Pallas; Coleoptera: Coccinellidae) by means of high throughput sequencing (HTS). For acquiring insights into diets of mobile flying insects at landscape scale minimizing trapping bias is important, which imposes methodological challenges for HTS. We therefore assessed the suitability of three field sampling methods (sticky traps, pan traps and hand-collection) as well as new aphid primers for identifying aphid prey consumption by coccinellids through HTS. The new aphid primers facilitate identification to species level in 75% of the European aphid genera investigated. Aphid primer specificity was high *in silico* and *in vitro* but low in environmental samples with the methods used, although this could be improved in future studies. For insect trapping we conclude that sticky traps are a suitable method in terms of minimizing sampling bias, contamination risk and trapping success, but compromise on DNA-recovery rate. The aphid diets of both field-captured ladybird species were dominated by *Microlophium carnosum*, the common nettle aphid. Another common prey was *Sitobion avenae* (cereal aphid), which got more often detected in *C*. *septempunctata* compared to *H*. *axyridis*. Around one third of the recovered aphid taxa were common crop pests. We conclude that sampling methodologies need constant revision but that our improved aphid primers offer currently one of the best solutions for broad screenings of coccinellid predation on aphids.

## Introduction

Insects, including crop pollinators and predators of crop pests, provide important ecosystem services to agriculture. Advancing our understanding of the dietary resource needs of service-providing insects is critical to effectively promote them by agricultural landscape management [1–3]. Various methods have been used to investigate diets of insects, each with distinct advantages and disadvantages [4]. High-throughput sequencing (HTS) has been increasingly adopted as a standard method for dietary analysis of both prey consumed by predators and plants consumed by herbivores, given its high accuracy [5] and its capacity to detect a broad range of consumed species simultaneously [6,7]. However, several methodological constraints remain for HTS-based dietary analyses, in particular with respect to the analysis of prey diets of insect predators. First, insects are small animals, which yield minute amounts of gut content, making it difficult to distinguish between contamination and actual prey consumed. Second, especially for this system, close taxonomic proximity between predators and prey makes it challenging to specifically amplify prey DNA, which is especially important since the entire animal is used, rather than just faecal samples. Furthermore, collecting large numbers of individual insect predators, from which prey DNA can be isolated and contamination avoided, is difficult. For example, widely-used approaches such as pitfall trapping, vacuum sampling or sweep-netting may ensure collection of insects in sufficient numbers and in satisfactory condition for DNA analysis [8–11], but these sampling methods can introduce cross-contamination through interaction of insects in the sampling containers [12–14]. Moreover, the resource-use patterns found in studies using such sampling methods are often prone to an “observer bias”, i.e. they may be dependent on the choice of sampling location. For example, if predators are hand-collected directly from easily accessible plants, samples may be biased towards prey associated with the sampled host plants and the very local habitat, rather than adequately representing dietary use or preferences of mobile insects in their entire foraging range. Sampling methods using traps that capture moving insects beyond the immediate trapping location, such as interception traps that capture insects during flight [15,16], or traps attracting insects over relatively large distances via colour, scent or light [16], could be more suitable for these reasons. However, also the use of trap-sampling methods presents challenges: for example, low insect sampling effectiveness during short trapping periods, or the risk of low DNA recovery rates due to DNA degradation if trapping periods are longer and thus restricted potential for DNA analysis [17,18]. Furthermore, in amplicon-based HTS analyses of diet, the choice of PCR primers is critical [9]. To increase amplification probability of target DNA, a primer pair should amplify as broad a range of potentially consumed food taxa as possible, whilst ideally not amplifying the consumer species itself [19]. Moreover, the amplicons generated should allow distinction of consumed taxa at an appropriate taxonomic resolution. For such studies, the primers need to target a gene with primer sites conserved between target species, while amplicons need to be sufficiently short to survive digestion but sufficiently long as to provide the required taxonomic information.

A group-specific aphid primer pair published by Harper *et al*. [20] promised amplification of a wide range of aphid species. However, it was not clear how well-suited it was for HTS, nor how well it might amplify, and distinguish between, different aphid species, and to what extent it also would amplify ladybirds and other arthropod taxa. Amplification performance of primers on environmental DNA can differ from *in silico* results and *in vitro* amplification of mock communities. For example, both of the latter methods showed the Clarke primers to be useful for DNA metabarcoding of insects [21,22]. In a study by Alberdi *et al*. [23], however, the same primers failed to amplify prey DNA from environmental samples, due to extensive amplification of non-target DNA. It is therefore important to test primer performance on real samples collected from the landscape. We focused on *Coccinella septumpunctata* (Linnaeus) and *Harmonia axyridis* (Pallas), two ladybird species (Coleoptera: Coccinellidae) known as key aphid predators in temperate agricultural landscapes [24,25]. While *C*. *septempunctata* is native to Europe, the Asian *H*. *axyridis* was introduced into European agricultural systems in the 1990s [26]. The role of *H*. *axyridis* as a natural enemy of crop pests motivated its introduction into many agroecosystems as a non-native biocontrol agent, from where it quickly spread and out-competed local ladybird populations [27,28]. Both ladybird species are amongst the most abundant ladybirds in the studied German and Swiss agricultural regions [29,30]. Their high functional importance as natural enemies of aphids has led to several prey choice and digestion studies under artificial conditions [31,32]. However, far less is known about aphid prey use of the two ladybirds in real agricultural landscapes. Yet, such knowledge is critical for targeted promotion of the two species as crop aphids’ natural enemies, as well as to inform management decisions with respect to the conflicting role of the invasive *H*. *axyridis* as pest control agent on one hand and predator or competitor with native insect species on the other hand. We therefore investigated the aphid prey of *C*. *septempunctata* and *H*. *axyridis*, compared the advantages and disadvantages of different trap and hand-collection based sampling approaches in terms of sampling effectiveness and aphid DNA detectability in ladybird guts. We modified the aphid-specific primer pair designed by Harper *et al*. [20] with respect to the applicability of HTS for investigating aphid prey use of functionally important ladybird species at the landscape scale. Specifically, we compared: (I) primer specificity between the existing and modified primer pairs as well as resolution of aphid identification; (II) the number of sampled ladybirds and their suitability for DNA analysis between sampling methods, and (III) aphid prey diets of field-sampled *C*. *septempunctata* and *H*. *axyridis*.

## Methods

### *In silico* and *in vitro* primer specificity

The Harper *et al*. [20] general aphid primer pair amplifies a region of 308 bp of the mitochondrial cytochrome *c* oxidase I subunit (COI) gene. To assess the suitability of the primer pair for this study, a sequence library was produced by downloading and clustering COI sequences of Coleoptera, Coccinellidae, Hemiptera, Hymenoptera, Aphididae, Neuroptera and Araneae from GenBank [33] via PrimerMiner v.0.18 [34]. Of these, Coccinellidae and Aphididae are directly relevant to the present study, whilst the other taxa were included to assess any broader potential of the modified primers for other studies. Sequences were aligned in Geneious Prime 2019.1.1. ([35] via MAFFT 1.3.7. [36]) and primer binding sites visually assessed on a subset of thirty species of aphids and coccinellids, represented by at least five sequences each. Subsequently, several modifications were made to the primer sequences to increase exclusion of ladybird DNA from amplification (Table 1), thus maximising recovery of prey reads [19]. PrimerMiner v.0.18 [34] was used to visualize differences in the alignment of both the modified primers and those designed by Harper *et al*. [20] to the target binding sites over the whole library (S1 Fig). The improvement of *in silico* primer target specificity was visualized and compared using PrimerMiner v.0.18 [34] with the default table for mismatch scoring and a penalty score of >120.

**Table 1:** Primers designed by Harper *et al*. [20] compared with those modified for this study.

Primer	Sequence (5’-3’)	Direction	Source	Tm [°C]	GC content	Molecular weight [g mol^-1^]
Aph344F	GGAACAGGWACAGGATGAAC	F	Harper *et al*. [20]	60.2	50%	6228.6
Aph149R	AATCAAAATAAATGTTGATA	R	Harper *et al*. [20]	49.5	15%	6156.2
Aph344.MF	GGAACAGGWACAGGATGAACWA	F	This study	62.6	45.5%	6850.6
Aph149.MR	AATCARAATARATGTTGATA	R	This study	49.2	20%	6172.1

The primers were further tested *in vitro* with DNA extracted from several ladybird, aphid and alternative predator specimens to approximately match those groups tested *in silico*, with particular focus on aphid diversity. These included ladybirds *C*. *septumpunctata* and *H*. *axyridis*, aphids *Aphis fabae*, *Myzus cerasi*, *Brachycaudus lychnidis*, *Sitobion avenae*, *Aphis rumicis* and *Microlophium carnosum*, and alternative predators *Chrysoperla carnea*, *Loricera pilicornis*, *Pardosa pullata*, Syrphidae *sp*. and Ichneumonidae *sp*. Extraction of DNA used Qiagen DNeasy Blood & Tissue Kits (Qiagen, Manchester, UK) following manufacturer instructions, but with an extended lysis time of 12 h for better penetration of chitinous insect tissue. Both primer pairs were tested in 5 μl reaction volumes comprised of 1 μl DNA, 2.5 μl PCR Multiplex Kit (Qiagen) and forward and reverse primers at 2 ng μl^-1^. All PCRs were carried out following: 95°C for 15 min, then 35 cycles of 94°C for 30 s, 51°C for 30 s and 72°C for 90 s, and a final extension of 72°C for 10 min. PCR products were visualised via gel electrophoresis in 2% agarose gels illuminated with UV light, the DNA stained with SYBR^**®**^Safe (Thermo Fisher Scientific, Paisley, UK).

Details were calculated using ThermoFisher’s Primer Analyzer. The primers designed by Harper *et al*. [20] were reported in an unconventional manner which has been corrected in this table to allow comparison with the new modifications.

### Taxon resolution of amplicon region

While primer specificity assessments need reference databases with broad taxon coverage, investigation of taxonomic resolution of a given amplicon mainly relies on correctly identified sequences. Especially in aphids, where morphological identification is sometimes impossible [37], it is difficult to obtain sequences from accurately identified specimens. Nevertheless, reference databases should be as comprehensive as possible to provide sufficient insight into both intra- and inter-specific variability. For this, the best currently available dataset for European aphids was used ([38]; It has been deposited on GenBank [33] (KF638720 to KF639739) and PhylAphidB@se website, http://aphiddb.supagro.inra.fr), which covers the full 658bp Folmer barcoding region [39] of COI. Aphid species expected in the study region, based on vegetation and aphid-host plant relations, were added to the library from GenBank. Library sequences not identified to species level and covering less than 296 base pairs of the amplicon region were excluded. The library produced contains 1160 sequences comprising 999 sequences from the aforementioned aphid database [38] and 161 additional sequences from GenBank (S1 Table), totalling 282 species across 95 genera. Sequences were aligned in ClustalX [40], manually checked in BioEdit [41] and trimmed in MEGA5 [42]. To assess aphid taxon assignability, a Blastn algorithm in Blast+ [43] with a clustering threshold of 90% was performed on the aforementioned library. After visually screening the matches the threshold was increased to 98.36% allowing a maximum of matches at species level while excluding deviating matches as often as possible. If matching sequences originated from the same species exclusively, a taxon was considered identifiable to species level. If several species matched, it was considered identifiable to genus level, since no incorrect matches occurred for this similarity threshold at higher taxonomic levels. This library was subsequently used as a reference database for aphid species identification of our field samples. The sequence similarity threshold informed on clustering thresholds necessary for centroid generation during bioinformatics processing of field samples (99%). This similarity threshold is rather high and leads to a high number of OTUs in ladybird taxa, which would allow taxon assignment with lower similarity thresholds.

### Study regions and ladybird sampling

Fieldwork was conducted in 2016 in agricultural landscapes of northern Switzerland (50 km radius around Zurich) and southern Germany (20 km radius around Landau, Pfalz) (S2 Table). A total of 23 independent agricultural landscape sectors of 500 m radius (hereafter landscapes) were chosen with different land use compositions. In each of the landscapes, five (Switzerland, 12 landscapes) or three (Germany, 11 landscapes) sampling points were randomly selected and equipped with two types of traps (sticky trap and combi trap, see below), adding up to a total of 186 traps. To minimize the risk of sampling non-target species of high conservation concern, traps were not set up in or near nature conservation areas. Trapping was in accordance with national legislation. We obtained permits for trapping in Germany from the “Struktur- und Genehmigungsdirektion Süd”, AZ 42/553-254 486/16. In Switzerland no permits were necessary, since no trapping was done in protected areas. Sampling points were located at least 200 m apart from each other. Ladybirds were sampled at each sampling point every two weeks from April to July, yielding eight sampling rounds (S3 Table). Combi traps are a combination of pan traps and intersection window traps, having two plexi-glass windows arranged cross-wise over a yellow funnel of 42.5 cm upper diameter [44] (S2 Fig). At the bottom of the funnel a whirl-pack^®^ bag (Sigma-Aldrich) was attached, filled with 95% ethanol, ensuring that captured ladybirds were preserved immediately after trapping. Each sticky trap consisted of two wooden plates (891 cm x 210 cm) painted with three lengthwise strips of UV-reflecting colour (yellow, blue, white; Sparvar UV reflecting colour of Spray-Color GmbH) for maximum attractiveness (S2 Fig). Transparent acetate foils (Folex Foils Laserptinter BG-64 from OfficeWorld Switzerland) were attached to the plates and sprayed with insect glue (Soveurode spray glue from Witasek, Austria). The foils and the bags were mounted at two week intervals and collected after four sampling days. This is a comparably long period for samples on sticky traps intended for genetic use, but it allows collection of sufficient numbers of individuals with a reasonable sampling effort [45]. In the 11 German landscapes, in addition to these two trap-sampling methods, habitats in the immediate surrounding of the sampling points were hand-sampled: ladybirds were collected with sweep nets from the vegetation of major habitat types present. All sampled *C*. *septempunctata* and *H*. *axyridis* were visually identified, collected into separate tubes filled with 95% ethanol and stored at -18°C until further processing.

### Laboratory procedures

To reduce PCR inhibitors in the ladybird bodies and to minimize the risk for potential contamination, elytra, wings, legs and heads of ladybirds were removed before DNA extraction. Isolation of ladybird guts was not possible due to disruption of internal tissue through storing in 95% ethanol. Extractions were performed with the QIAGEN^**®**^ Frozen Plant Tissue (DNeasy 96) kit on a total of 619 ladybirds following homogenisation with a QIAGEN^**®**^ TissueLyser II bead mill (Qiagen, Manchester, UK). On each extraction plate (96 samples) three to six negative controls were included. The tubes assigned for negative control were treated precisely as any other sample starting from DNA extraction throughout all laboratory steps until visualisation of the PCR product. For aphid DNA amplification, the modified primers detailed above were used (Table 1). Molecular identifier tags (MID-tags) were attached to both primer pairs so that individual ladybirds could be identified after pooling during bioinformatic processing. The PCR reaction volume of 6.5μl consisted of 3.125μl Multiplex mix (Quiagen) and 0.125μl primer solution per primer, yielding a concentration of 10pmol/μl primer plus 2.125μl water and 1μl DNA per reaction tube. All PCRs took place in a GeneAmp9700 PCR system performing the following cycles: 95°C for 15min, 40 x (94°C for 30s, 51°C for 90s, 72°C for 90s) and a terminal phase of 72°C for 10min. PCR cycling conditions were optimized using PCR temperature gradients followed by examining the intensity of the PCR product after gel electrophoresis. Gel electrophoresis was run in a 2% agarose gel in Tris-acetate-buffer (TAE) running for 40min at 140 Volt, stained with 0.5 mg ml^-1^ SYBR^**®**^Safe (Thermo Fisher Scientific, Paisley, UK) to identify successful PCR amplification and to monitor possible contamination of negative controls included in the samples. All samples yielding a positive PCR product were quantified by Qubit measurements (ThermoFisher Scientific, Waltham, MA, UK) and pooled equimolarly into two pools to ensure sufficient read depth for sequencing. The pools were purified with SPRIselect (© 2012 Beckman Coulter, Inc.; left side selection with a ratio of 0.8 for both pools) to remove primer dimer and then further processed with the NEXTflex^**®**^ Rapid DNA-Seq Kit from BiooScientific for library building. HTS was performed with an Illumina MiSeq Sequencer at the Genomics Research Hub at Cardiff University School of Biosciences using a MiSeq Reagent Kit v3 from Illumina (600 cycles with 2 x 300 bp). Raw MiSeq data for all samples described in the manuscript have been uploaded to NCBI Sequence Read Archive under SRA Accession number PRJNA563315. Information on bioinformatics procedure can be found in the bioinformatics section below and in the (S1 Appendix) as well as detailed individual-level taxonomic data in the file S1 Data.

### Bioinformatics

Paired-end Illumina reads were filtered for quality using Trimmomatic v0.32 [46]. The command ILLUMINACLIP:TruSeq3-PE-2.fa:2:30:10 was used to remove adapters. Leading and trailing low quality bases were removed if their quality score was below 3. A minimum length of 250 bp and a minimum average base quality score of 20 over a sliding window of four bases were specified. Filtered reads were then aligned using FLASH v1.2.11 [47]. The trim.seqs command was used in Mothur v1.37.1 [48] to assign reads to their respective sample identifications based on MID tag sequence combinations (with S5_P1Oligos.txt and S5_P2Oligos.txt for the respective pools, located in the S2 and S3 Files), and allowing for one mismatch, prior to MID tag and primer removal. Subsequently, reads were demultiplexed into one file per sample using bespoke perl scripts (S1 Appendix; Demultiplexing). Chimeric sequences alongside those appearing fewer than 10 times in a single sample were removed using the unoise2 and minuniquesize commands in Usearch v9.2.64 [49]. This threshold of 10 was later adjusted to 13 for pool1 and 97 for pool2 as a method to mitigate for tag-jumping, contamination or sequencing errors following Dunn *et al*. [50] (S1 Appendix; Mitigating tag-jumping). Usearch v9.2.64 was also used to cluster similar sequences into centroids using an identity threshold of 99% utilising the cluster_fast algorithm. The header line for each centroid was then annotated with the sample identification before concatenating all centroids into a single file ready for taxonomic assignment. The Blastn algorithm in Blast+ [43] was used for taxonomic assignment against the library described above for analysis of taxonomic resolution. Blastn parameters were identical to the ones used for identification of taxon resolution in the region amplified i.e. a minimum read length of 296 bp and a minimum sequence similarity of 98.36%. For centroids that did not match to the library, a Blastn search was performed on GenBank.

### Statistical analysis

Differences in sampling effectiveness (i.e. the number of captured ladybird individual per trap and sampling interval) of the two trap types applied (sticky traps and combi traps) were analysed by running generalised linear mixed models (GLMM) with Poisson error distributions using R package lme4 v1.1–17 [51]. Models included trap type, ladybird species (*C*. *septempunctata* and *H*. *axyridis*) and their interaction as fixed factors as well as country, landscape and sampling point as nested random factors with 4 sampling intervals as random slope. Sampling intervals comprised two pooled sampling rounds of a four day duration each, with sampling effort standardised between the two trap types. DNA recovery rate (presence-absence data; i.e. the number of ladybird individuals in which aphid DNA was detected (presence) or not detected (absence) using a certain sampling method at a sampling point during a sampling interval) was compared between hand-sampled ladybirds and trap-sampled ladybirds. Samples from the two trap types (sticky traps and combi traps) were pooled in this model since no significant differences in recovery rate were detected (not shown). A GLMM with binomial error distribution and the same random structure as described above was run. In both models log-likelihood ratio tests were used for statistical inference [52]. To explore differences in prey species used by *C*. *septempunctata* and *H*. *axyridis*, multivariate differences in detected consumed aphid species composition were assessed using the adonis function implemented in the R package vegan (2.5–2) [53]. The adonis function is applied on distance measures derived from a matrix, which in this case contains proportions of detected aphid species per landscape per sampling round. The matrix was Hellinger-transformed to deal with the relative data type and the high zero-ratio [54] before Euclidean distances were calculated. The adonis function included ladybird species as factor using sampling round as stratum on the 999 permutations performed, so differences in aphid species composition would not interfere with differences between sampling rounds. Visualisation of the data was performed with non-metric multidimensional scaling (NMDS) of Hellinger-transformed Euclidean distances with k = 2, using the metaMDS function. All statistical analysis were performed in R version 3.4.1 [55].

## Results

### *In silico* and *in vitro* primer specificity

**Fig 1:**
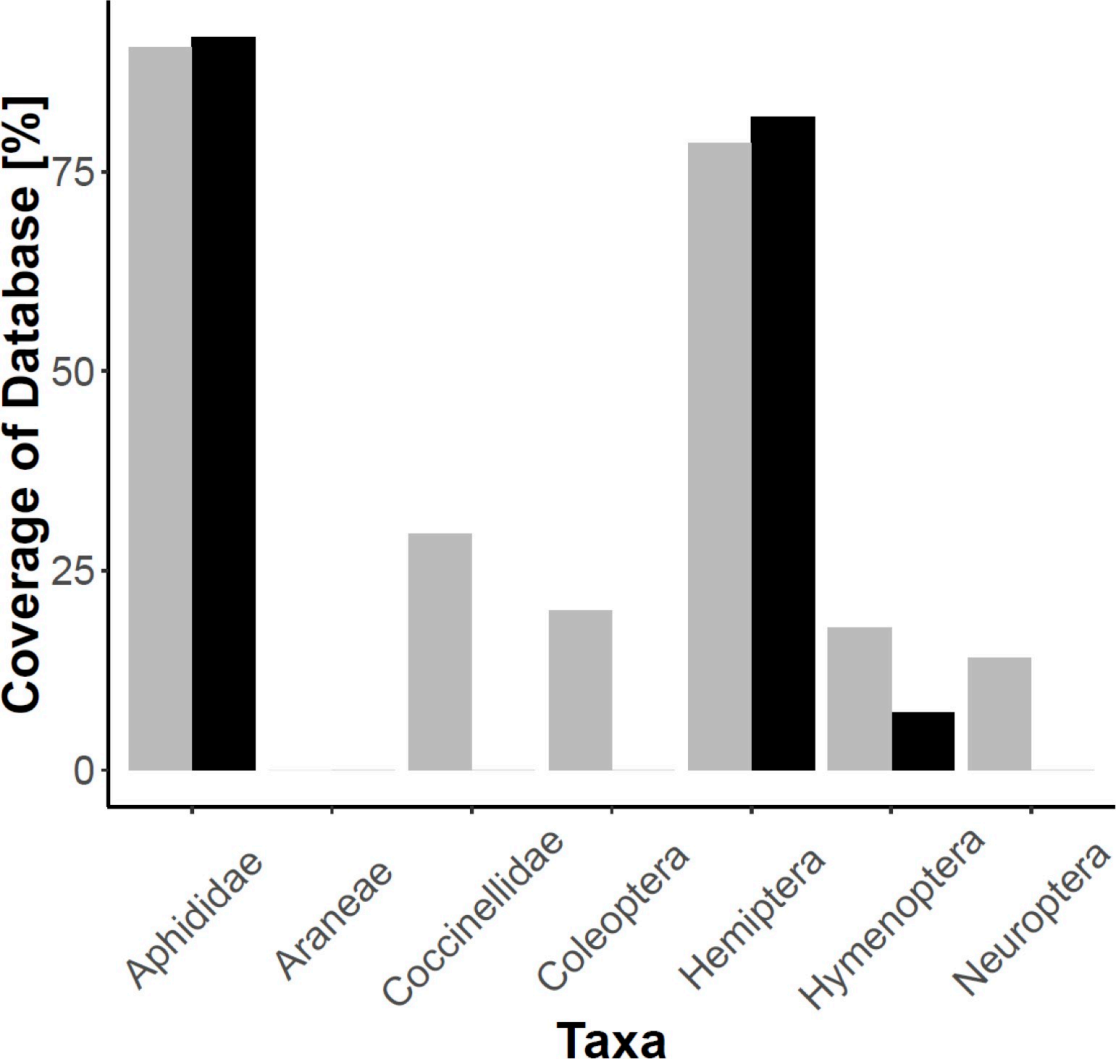
Percentage coverage by the two primer pairs for different taxa. The new modified primers designed in this study (black) and those of Harper *et al*. [20] (grey).

Alignments displayed clear mismatches between the primer sequences designed for this study and ladybird sequences (S1 Fig). *In silico* evaluation of the primers designed by Harper *et al*. [20] suggested successful amplification of 90.61% of aphids and 29.55% of coccinellids. The primers modified for this study, however, successfully amplified 91.78% of aphids and 0% of coccinellids. The modified primer pair achieved increased amplification for Hemiptera generally and, other than a relatively low percentage of Hymenoptera, did not amplify any of the other predatory groups evaluated (Fig 1). These results were ratified in the *in vitro* tests (S4 Fig), with the Harper *et al*. primers achieving broad amplification success with only the Ichneumonid wasps not amplifying, although some of the ladybirds and alternative predators were amplified faintly. The modified primers, however, amplified all aphids (one slightly fainter) but none of the ladybirds or alternative predators.

### Taxon resolution of amplicon region

All 1,160 aphid sequences investigated from GenBank were assignable to genus level with a sequence similarity of 98.36% or better. Of the 32 genera investigated, 24 allowed taxon resolution to species level, covering a total of 69 species. In eight genera, taxonomic resolution to species level was not possible (*Aphis*, *Betulaphis*, *Brachycaudus*, *Dysaphis*, *Macrosiphonella*, *Macrosiphum*, *Uroleucon*, *Wahlgreniella*). Intra-specific similarity was 99.81% ± 0.05 for all species represented by two sequences or more. Within-genus variability was calculated for sequences that could only be identified to genus level and which were represented by more than two taxa per genus. Their average sequence similarity was 99.74 ± 0.23%.

### OTUs retrieved from field samples

Initial read numbers following HTS were 5,668,854 and 2,772,817 from the first and second sequencing runs, respectively, resulting in an average of 6,348 and 8,531 reads per sample in each pool. After removing adaptors and low quality reads with Trimmomatic v0.32, 1,639,236 and 1,300,972 reads remained. Following alignment with FLASH v1.2.11, 1,622,258 and 1,279,716 reads were retained. Finally, 1,258,164 and 515,858 sequences remained after pairing aligned sequences with their respective MID tags in Mothur v1.37.1. Of the 141 OTUs (molecular operational taxonomic units) retrieved from analysed ladybirds, 43 could be assigned to aphid DNA sequences in the library and 89 were assigned to ladybirds (S1 Data). Eight OTUs did not match any sequence in the library and therefore a Blastn search was performed on GenBank. One further OTU could be assigned to the aphid *Laingia psammae* uniquely matching with more than 99% occurring in one ladybird individual. Resulting read numbers added up to 83,815 reads for aphids and 848,353 reads for ladybirds (see also OTU rarefaction curve S3 Fig). Given the 0% amplification of ladybirds in the *in silico* and *in vitro* tests, the ladybird read proportion found in field samples is rather high. A total of 21 aphid genera were found in ladybirds. Four taxa (*Aphis*, *Brachycaudus*, *Macrosiphum*, *Wahlgreniella*) could only be assigned to genus level. A total of 20 aphid species were distributed over the 17 other genera retrieved from ladybird guts. *Microlophium carnosum* and *Aphis* spp. were the most common taxa identified. They exhibited both the highest read numbers (45,492 and 15,057, respectively) and the highest frequency in ladybird guts (found in 51.1% and 22.6% of ladybirds positive for aphids, respectively) (S4 Table, S1 Data).

### Comparison of field sampling methods

**Fig 2:**
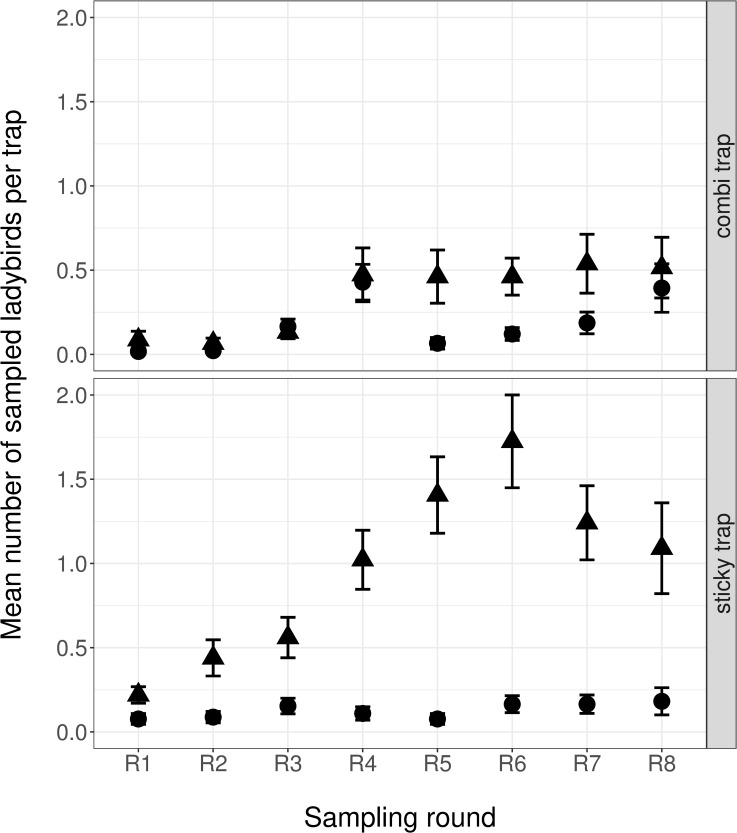
Mean (± SE) numbers of sampled ladybirds with the two trap types (combi traps and sticky traps). Circles denote *C*. *septempunctata* and triangles denote *H*. *axyridis*. Sampling rounds indicate two-week sampling intervals from April to July. See methods section for detailed description of trap types and sampling design.

A total of 1,040 *C*. *septempunctata* and *H*. *axyridis* were sampled with the two trap-sampling methods (S2 Data). With 720 (average per trap = 0.53 ±0.04) individuals in total, sticky traps yielded significantly more ladybirds than combi traps (320 individuals, average per trap = 0.25 ±0.02). Significantly more *H*. *axyridis* (854) than *C*. *septempunctata* (186) were captured. According to an interactive effect of trap type and ladybird species, the representation of *H*. *axyridis* was stronger in sticky traps (88.6% of individuals) than in combi traps (67.5% of individuals; Fig 2, Table 2). Hand-collections in Germany yielded more ladybirds than trap sampling, yielding 237 *C*. *septempunctata* and 359 *H*. *axyridis* individuals.

**Table 2:** Statistical evaluation of trapping success and DNA recovery rates.

Response	Fixed effects	*df*	*Χ*^*2*^	*p-value*
a) Ladybird trapping effectiveness	Trap type x ladybird species	1	62.7	< 0.001
	Ladybird species	1	464.9	< 0.001
	Trap type	1	155.8	< 0.001
b) Aphid DNA recovery rate from ladybird guts	Trap type x ladybird species	1	0.7	0.413
	Ladybird species	1	6.5	0.011
	Sampling method	1	36.8	< 0.001

Statistical inference using log-likelihood ratio tests for generalized linear mixed-effect models to test for differences in (a) ladybird trapping effectiveness of the two trap types (“trap type”; combi trap vs. sticky trap) for the two ladybird species (*C*. *septempunctata* and *H*. *axyridis*), and (b) aphid DNA recovery rates from ladybird guts for hand-sampled vs. trap-sampled ladybirds (combi traps and sticky traps combined; “sampling type”) for the two ladybird species. See Methods section for detailed description of sampling design and methods, and statistical analyses.

### Aphid DNA recovery

Genetic analyses were performed on a subset of 619 ladybirds (213 *C*. *septempunctata* and 406 *H*. *axyridis*), the remaining samples were used for palynological analyses published elsewhere [56]. Of those analysed here, 330 were hand-sampled and 289 were sampled with traps. Aphid DNA was detected in 186 ladybirds. Of the hand-sampled ladybirds, 167 were positive for aphids consisting of 82 *C*. *septempunctata* and 85 *H*. *axyridis*. A total of 19 ladybirds, from which aphid DNA was recovered, were trap-sampled, consisting of 12 *H*. *axyridis* and 7 *C*. *septempunctata*. DNA recovery rate in hand-sampled ladybirds was almost eight times higher (50.6%) than in those sampled with traps (6.6%). In addition, aphid DNA recovery was higher in *H*. *axyridis* (41.7%) than in *C*. *septempunctata* (23.9%) (Table 2, S1 Data).

### Ladybird diet

**Fig 3:**
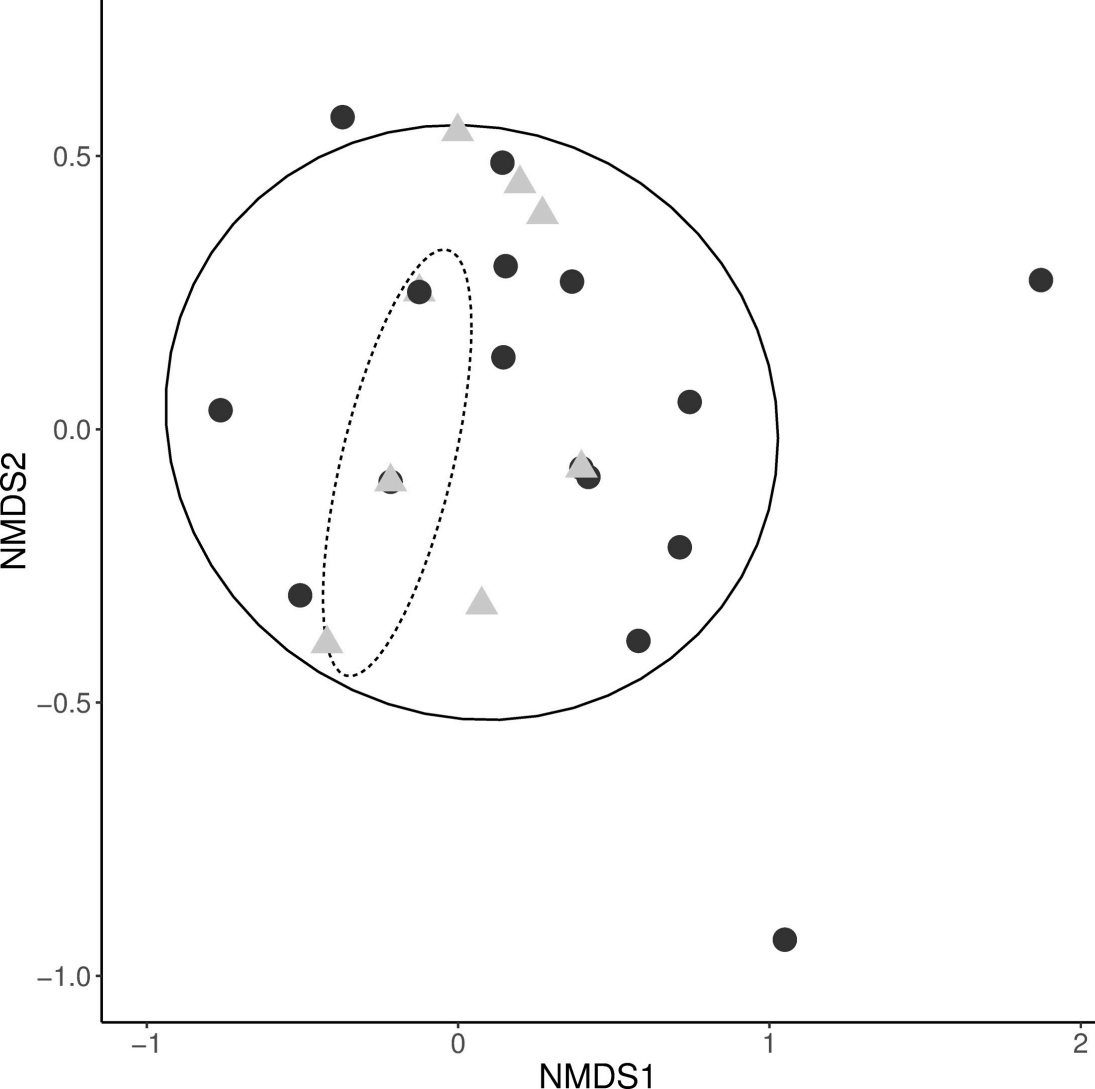
NMDS ordination graph for the two hand-sampled ladybird species. Comparing aphid prey species composition. Full line with points are *C*. *septempunctata*, grey triangles with the dashed line are *H*. *axyridis* (stress = 0.06), circles indicate a 95% confidence interval.

**Fig 4:**
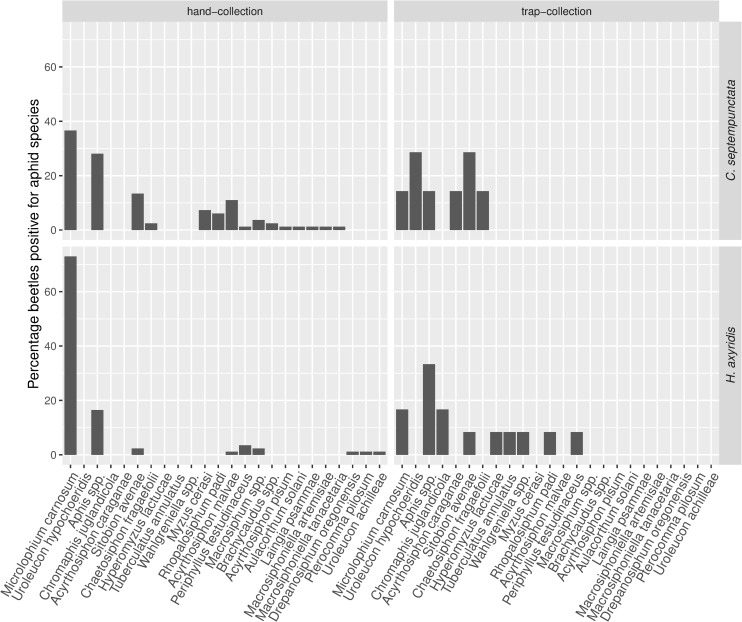
Aphid prey species consumed by the two studied ladybird species. Total number of hand-sampled ladybirds positively tested for aphids was 167 compared to 19 trap-sampled ladybirds (see S1 Data for more detail).

The diet of hand-sampled *C*. *septempunctata* was more variable than that of *H*. *axyridis* (multivariate dispersion: *F* = 12.03, *P* = 0.005). Despite some overlap in the species composition of aphids consumed by the two ladybird species the analysis revealed significant differences in aphid species compositions consumed by *C*. *septempunctata* and *H*. *axyridis* (Fig 3, *F* = 4.67, *P* = 0.020). *Microlophium carnosum* (stinging nettle aphid found in 88 ladybirds) and *Aphis* spp. (found in 25 ladybirds) were the most common taxa consumed by both ladybird species. However, *M*. *carnosum* was more often consumed by *H*. *axyridis* (72.9%) than by *C*. *septempunctata* (36.6%, Fig 4, S1 Data). For *C*. *septempunctata*, *Aphis* spp. and *S*. *avenae* (cereal aphid) comprised a greater fraction in the diet (28.0% and 13.4%) compared to *H*. *axyridis* (16.5% and 2.4%, Fig 4). All other aphid taxa were only found in a few ladybird individuals. Numbers of trap-sampled ladybirds positive for aphids were too low (a total of 19 individuals) for statistical comparison of ladybird prey. While hand-sampled ladybirds were positive for 18 aphid taxa, the 19 trap-sampled ladybirds were positive for 12 taxa, of which six were found in trap-sampled ladybirds exclusively (Fig 4). Thus, the most commonly consumed aphid taxa by hand-sampled ladybirds could also be found in trap-sampled individuals.

## Discussion

Our results allow insights into the aphid diet of two of the functionally most important ladybird species of Central European agricultural landscapes, *C*. *septempunctata* and *H*. *axyridis*, as well as the methodological possibilities and challenges of HTS as a means for investigation of dietary use of insects in real landscapes. Evaluation of the modified primer pair showed promising results *in silico* regarding both coverage and specificity, further ratified *in vitro* with the modified primers showing far greater specificity for aphids. A wide range of aphid taxa were also amplified from field-sampled ladybirds, although with a loss in specificity. The increased predator amplification for gut content samples could suggest that the identifying tags used in this study increased predator amplification, which could be avoided by attachment of tags with the sequencing adapters rather than before the PCR stage, although this could be associated with different biases. The amplicon region proved suitable for aphid identification to species level in most taxa, allowing insights into dietary use of hand-sampled ladybirds. Trap-sampling was mainly subject to low DNA recovery rates, yielding too few beetles positive for aphids for robust statistical analysis of prey composition, despite reasonable trapping numbers in sticky traps. *M*. *carnosum* and *Aphis* spp. were identified as the most frequently consumed prey in hand-sampled *C*. *septempunctata* and *H*. *axyridis*, and were also common in trap-sampled individuals.

### Primer suitability

*In silico* and *in vitro* evaluation of both the primers designed by Harper *et al*. [20] and the novel modifications from this study demonstrated improved specificity achieved by the modified primers. The modified primers achieved slightly larger coverage of aphids with greatly reduced amplification of coccinellids. The lack of amplification of many common agricultural predator groups such as spiders and ground beetles also suggests that the modified primers may be more broadly applicable to the HTS-based investigation of aphid predation by other species. The proportion of 94% predator reads recovered in our study is certainly at the high end of predator read proportions found in invertebrate predator studies [9]. Nevertheless, *in silico* and also *in vitro*, tests provide an insight into the potential bias against specific taxa by primers during the PCR process. That the modified primers had a 0% success rate with coccinellids *in silico* indicates a strong bias against them in PCR, likely resulting in a greater proportion of prey DNA reads post-sequencing than would be achieved with the primers designed by Harper *et al*. [20] regardless of predator amplification.

Instances in which ladybird DNA, other than that of the predator respective to each sample, was identified may indicate intraguild predation. Ladybirds are known to engage in intraguild predation, particularly consumption of other ladybird eggs and larvae [57]; whilst this is unarguably of agricultural significance, it was beyond the remit of this study, but highlights the possibility of future investigations of this aspect of ladybird biocontrol dynamics. Equally, co-occurrence of different ladybirds in samples could indicate cross-contamination of ladybirds during trapping. Attempts were, however, made to mitigate risk of this by removal of external wing cases and other non-essential body parts, indicating a greater likelihood of the aforementioned intraguild predation. When amplifying DNA of the predator in dietary metabarcoding, the high volume of predator reads generated could also increase the rate of tag-jumping and misassignment between samples, which may result in predator reads appearing in other samples. Whilst this would be problematic for broader dietary studies, the focus of this study on aphid predation circumvents the issue, although it is certainly worth considering for future studies pertaining to intraguild predation.

Taxonomic identification for this amplicon region was possible down to species level in 75% of European aphid genera based on the library used. This is good considering the relatively short amplicon length of 308 bp and the often cryptic taxonomy of aphids [58], though resolution may be weaker on a global scale. The taxa for which resolution was lower also proved difficult to identify to species level both morphologically and genetically, even when using the entire Folmer COI barcoding region [38]. It is questionable whether any sequence fragment in a size suitable for gut content analysis could facilitate better taxonomic resolution within COI. Other aphid specific primer pairs have been reported for the ribosomal 18S and mitochondrial COII barcoding regions [59,60], but both markers did not provide enough reference data on NCBI or BOLD to even identify all species found in ladybird guts analysed here. Currently, there seems to be a trade-off for broad taxonomic assessments between ribosomal and mitochondrial barcoding regions. It is not yet clear which region provides better taxonomic resolution [21], but currently 16S primer sets seem to give better taxonomic coverage, amplifying taxa more evenly, while far more sequence information is available for COI [21,22]. An extensive reference database for sequence identification is a prerequisite for any study aiming at obtaining a realistic insight into the dietary habits of its study organisms. Especially on a global scale, barcode availability for any aphid amplicon region is still low. The last count by Lee *et al*. [61] of the approximately 5000 species of aphids described in the world revealed that only 10% had a barcode on one of the commonly used platforms (BOLD or NCBI), which imposes major limitations to HTS.

### Trapping methods and DNA recovery

Sticky traps were more effective in trapping ladybirds than combi traps in the present study. However, DNA recovery was similarly limited for both trap types, yielding very few individuals testing positive for aphid DNA in the ladybirds’ guts. Hand-sampling seems more effective in this respect, with a DNA recovery rate almost eight times higher than in trap-sampled ladybirds. Even though sticky traps did not yield sufficient data to statistically evaluate aphid prey composition, they do provide valuable information, putting results of hand-sampled ladybirds into perspective. The dominant species found in hand-sampled ladybirds were found in trap-sampled ladybirds as well. Of the 12 taxa identified in trap-sampled ladybirds, six were not found in hand-sampled ladybirds, however. Remarkably, two of these species feed on high stemmed trees exclusively, which are difficult to access when hand-sampling: *Chromaphis juglandicola* on *Juglans regia* (Walnut) and *Tuberculatus annulatus* on *Quercus* spp. (Oak). In contrast, none of the aphid species detected in hand-sampled ladybirds are specific to high stemmed trees. Another noticeable result concerns the number of aphid taxa recovered by trap-sampling and hand-sampling. The ratio of aphid taxa found in ladybirds relative to the number of ladybirds testing positive for aphids was six times higher in trap-sampled ladybirds (0.63) than in hand-sampled ladybirds (0.11). This indicates that diet information derived from hand sampling is biased towards low numbers of species. Given the eight times higher aphid DNA detection rate in hand-sampled individuals, the use of traps might seem to be a high price to pay for a less biased, broader insight into predator diets. However, DNA recovery rates from trapped ladybirds have the potential to be improved (e.g. with shorter trap activity periods), while minimized sampling bias is crucial to inform on ladybird diet at the landscape scale. Taking these findings together, they suggest that sticky trap-sampling likely gives more representative insights into ladybird diet by considerably reducing sampling bias towards the sampled local vegetation. A further advantage of sticky trap-sampling is the reduction in potential cross-contamination, reported to be problematic in methods allowing interactions of trapped insects in the sampling container, such as in combi traps, sweep-netting, beating or vacuum sampling [12–14]. Thus, there are some limitations for this method mainly imposed by trade-offs between sampling effectiveness, DNA recovery rate and sampling effort. DNA detectability half-lives are influenced by many factors but were generally found to be less than a day for aphids in arthropod predator guts [20,59]. The detection rates on field samples yielded by our primers were unknown but sufficient trapping rates were a prerequisite for any aphid DNA detection and were ensured by elongating trapping periods to four days [45]. In future we recommend daily sample collection from sticky traps with more sampling rounds and/or traps to ensure sufficient sample size. This comes at a cost of higher sampling effort, but appears necessary for a representative picture of predator diet.

### Ladybird diet

Of the 18 aphid taxa identified in the 167 hand-sampled ladybirds’ guts, six were prey taxa shared between *C*. *septempunctata* and *H*. *axyridis*. *M*. *carnosum* was consumed by almost twice as many *H*. *axyridis* as *C*. *septempunctata*; nevertheless, it was the main prey found in both ladybirds. Similarly, the percentage of recovered pest taxa was comparable between ladybird species (33.4% and 46.7%, respectively), though, unlike *H*. *axyridis*, *C*. *septempunctata* consumed clearly more *S*. *avenae*, which aligns with the findings of Honěk *et al*. [62], who highlighted the association between *C*. *septempunctata* and cereal crops. Most other taxa were only detected in a relatively low number of ladybird individuals in either ladybird species, with limited overlap between *C*. *septempunctata* and *H*. *axyridis*. Accordingly, prey composition differed significantly between the two ladybird species. Several reasons are discussed for the rapid increase in dominance by the invasive *H*. *axyridis* in European ladybird communities, mainly intraguild predation, apparently common in *H*. *axyridis* [63], and food resource competition [64,65]. Shared use of frequently consumed aphid preys, which are specialised on a specific host plant species, as shown in our study, certainly increases the potential for resource competition between the two studied ladybird species. This makes *C*. *septempunctata* vulnerable to competition and intraguild predation by *H*. *axyridis* and may be a reason why *H*. *axyridis* so strongly dominates local ladybird populations as recorded in this and other studies [65,66]. Despite these results according with previous findings, a potential observer bias present in the hand-collected ladybird samples should be considered, given that an effect by choice of the local sampling vegetation cannot be excluded here. For this reason, future improvement of trap-based sampling methods is important.

### Conclusions and implications

This study highlights some important methodological challenges, but also presents potential solutions towards improved sampling, when using HTS for investigations of prey use by insect predators at the landscape scale. Our modified primers gave us insights into aphid prey use by ladybirds, amplifying a wide range of aphid taxa that could be identified to species level. The primer set used for this study still has restrictions in both specificity and taxon resolution, but, as long as insufficient reference data are available for primers situated in more suitable barcoding regions, we think that this primer is probably among the best current solutions for broad taxonomic screenings for aphids in ladybird guts.

For acquiring insights into diets of mobile flying insects at scales beyond local vegetation (e.g. at the landscape scale), we recommend sticky traps for future investigations. Our findings indicate a more complete spectrum of prey taxa retrieved, including taxa from a broader range of habitats likely used by prey that are not usually accessible via hand-sampling. Our findings regarding the species composition of the consumed aphids by the invasive *H*. *axyridis* compared to the native *C*. *septempunctata* indicate significant dissimilarities in prey communities, but also several shared aphid prey, including host-specific species. The latter thus provides some support with real agricultural landscape data on resource competition between the invasive and the native species, as has been suggested by previous studies. The dominance of nettle aphids in both ladybird species underlines the role of nettle as an important source habitat of beneficial insects in agricultural landscapes.

## Supporting information

S1 Table Library for aphid taxon resolution assessments. (DOCX)

S2 Table Central coordinates of landscape sectors. (DOCX)

S3 Table Sampling rounds and corresponding dates. (DOCX)

S4 Table Aphid OTU and species information retrieved from ladybird samples. (DOCX)

S1 Fig Visualisation of primer binding sites. Primers modified for this study and those used by Harper *et al*. (2005) aligned with mass-alignments of taxa downloaded via PrimerMiner. The forward primer is on the left, the reverse primer on the right, both sequence alignments are oriented in 5’– 3’ direction.
(TIF)

S2 Fig Sampling point with the two trap types. The combi trap (yellow, 42.5 cm upper diameter) has a whirl-pack^®^ bag (Sigma-Aldrich) attached on the bottom, filled with 95% ethanol and a roof on top to prevent dilution through rain. For the sticky trap (891cm x 210cm), foils (Folex Foils Laserptinter BG-64 from OfficeWorld Switzerland) are attached with clothes pegs and/or tape to the coloured board (yellow, blue, white; Sparvar UV reflecting colour of Spray-Color GmbH) and were then sprayed with glue (Soveurode spray glue from Witasek, Austria).
(TIF)

S3 Fig OTU rarefaction curve over ladybird samples. Curvature index: y = 0.5826x^0.675^ on 619 samples.
(TIF)

S4 Fig *In vitro* comparison of primers. The modified primers of this study show increased specifity towards target aphid DNA, missing to amplify ladybird DNA.
(TIF)

S1 Data Analysed ladybirds with aphid OTU and read information. (XLSX)

S2 Data Trapped ladybirds. (XLSX)

S1 Appendix Bioinformatics details. (DOCX)

S1 File File for deplexing pool1. (TXT)

S2 File File for deplexing pool1. (TXT)

S3 File File for deplexing pool2. (TXT)

S4 File File for deplexing pool2. (TXT)

S5 File File for deplexing pool1. (TXT)

S6 File File for deplexing pool2. (TXT)
