# Supplementary material for: Insights into aphid prey consumption by ladybirds: Optimising field sampling methods and primer design for high throughput sequencing
Source: PLoS One. 2020 Jul 1;15(7):e0235054. doi: 10.1371/journal.pone.0235054 (PMC7329105; doi:10.1371/journal.pone.0235054)
Supplement: S1 Table — (DOCX) [file pone.0235054.s001.docx]

**S1 Table. Library for aphid taxon resolution assessments.**

| Species | Accessory number | Source |
| --- | --- | --- |
| *Cavariella aegopodii* | ACEA001-14 | BOLD |
| *Hyperomyzus lactucae* | ACEA002-14 | BOLD |
| *Myzus varians* | ACEA003-14 | BOLD |
| *Cavariella theobaldi* | ACEA004-14 | BOLD |
| *Chromaphis juglandicola* | ACEA005-14 | BOLD |
| *Macrosiphum albifrons* | ACEA006-14 | BOLD |
| *Phorodon humuli* | ACEA007-14 | BOLD |
| *Eucallipterus tiliae* | ACEA008-14 | BOLD |
| *Cavariella theobaldi* | ACEA009-14 | BOLD |
| *Cavariella theobaldi* | ACEA010-14 | BOLD |
| *Eriosoma lanuginosum* | ACEA011-14 | BOLD |
| *Megoura viciae* | ACEA012-14 | BOLD |
| *Amphorophora rubi* | ACEA013-14 | BOLD |
| *Brachycaudus cardui* | ACEA014-14 | BOLD |
| *Myzus lythri* | ACEA015-14 | BOLD |
| *Myzocallis coryli* | ACEA016-14 | BOLD |
| *Brachycaudus cardui* | ACEA017-14 | BOLD |
| *Ovatus crataegarius* | ACEA018-14 | BOLD |
| *Callipterinella tuberculata* | ACEA019-14 | BOLD |
| *Semiaphis dauci* | ACEA020-14 | BOLD |
| *Macrosiphum cholodkovskyi* | ACEA021-14 | BOLD |
| *Myzus lythri* | ACEA022-14 | BOLD |
| *Myzus lythri* | ACEA023-14 | BOLD |
| *Thelaxes dryophila* | ACEA024-14 | BOLD |
| *Aphis grossulariae* | ACEA025-14 | BOLD |
| *Aphis ulmariae* | ACEA026-14 | BOLD |
| *Brachycaudus cardui* | ACEA027-14 | BOLD |
| *Metopeurum fuscoviride* | ACEA028-14 | BOLD |
| *Thecabius affinis* | ACEA029-14 | BOLD |
| *Macrosiphoniella absinthii* | ACEA030-14 | BOLD |
| *Aphis verbasci* | ACEA031-14 | BOLD |
| *Aphis farinosa* | ACEA032-14 | BOLD |
| *Brachycaudus cardui* | ACEA033-14 | BOLD |
| *Nasonovia ribisnigri* | ACEA034-14 | BOLD |
| *Anuraphis shaposhnikovi* | ACEA035-14 | BOLD |
| *Aphis verbasci* | ACEA036-14 | BOLD |
| *Aphis epilobii* | ACEA037-14 | BOLD |
| *Aphis epilobii* | ACEA038-14 | BOLD |
| *Cinara laricis* | ACEA039-14 | BOLD |
| *Cinara pectinatae* | ACEA040-14 | BOLD |
| *Rhopalosiphum insertum* | ACEA041-14 | BOLD |
| *Uroleucon hypochoeridis* | ACEA042-14 | BOLD |
| *Uroleucon jaceae* | ACEA043-14 | BOLD |
| *Melanaphis luzulella* | ACEA044-14 | BOLD |
| *Uroleucon solidaginis* | ACEA045-14 | BOLD |
| *Aphis teucrii* | ACEA046-14 | BOLD |
| *Macrosiphum sileneum* | ACEA047-14 | BOLD |
| *Aphis salicariae* | ACEA048-14 | BOLD |
| *Baizongia pistaciae* | ACEA049-14 | BOLD |
| *Aphis fabae* | ACEA050-14 | BOLD |
| *Aphis verbasci* | ACEA051-14 | BOLD |
| *Cavariella theobaldi* | ACEA052-14 | BOLD |
| *Cinara brauni* | ACEA053-14 | BOLD |
| *Uroleucon picridis* | ACEA054-14 | BOLD |
| *Uroleucon sonchi* | ACEA055-14 | BOLD |
| *Macrosiphoniella abrotani* | ACEA056-14 | BOLD |
| *Uroleucon jaceae* | ACEA057-14 | BOLD |
| *Brevicoryne brassicae* | ACEA058-14 | BOLD |
| *Hyadaphis foeniculi* | ACEA059-14 | BOLD |
| *Dysaphis plantaginea* | ACEA060-14 | BOLD |
| *Aphis davletshinae* | ACEA061-14 | BOLD |
| *Aphis hederae* | ACEA062-14 | BOLD |
| *Ceruraphis eriophori* | ACEA063-14 | BOLD |
| *Ceruraphis eriophori* | ACEA064-14 | BOLD |
| *Aphis fabae* | ACEA065-14 | BOLD |
| *Aphis sambuci* | ACEA066-14 | BOLD |
| *Aphis hederae* | ACEA067-14 | BOLD |
| *Aphis fabae* | ACEA068-14 | BOLD |
| *Aphis fabae* | ACEA069-14 | BOLD |
| *Aphis fabae* | ACEA070-14 | BOLD |
| *Aphis fabae* | ACEA071-14 | BOLD |
| *Aphis fabae* | ACEA072-14 | BOLD |
| *Aphis fabae* | ACEA073-14 | BOLD |
| *Aphis fabae* | ACEA074-14 | BOLD |
| *Aphis fabae* | ACEA075-14 | BOLD |
| *Aphis fabae* | ACEA076-14 | BOLD |
| *Aphis fabae* | ACEA077-14 | BOLD |
| *Aphis fabae* | ACEA078-14 | BOLD |
| *Aphis fabae* | ACEA079-14 | BOLD |
| *Aphis fabae* | ACEA080-14 | BOLD |
| *Aphis fabae* | ACEA081-14 | BOLD |
| *Aphis fabae* | ACEA082-14 | BOLD |
| *Aphis newtoni* | ACEA083-14 | BOLD |
| *Aphis fabae* | ACEA084-14 | BOLD |
| *Aphis fabae* | ACEA085-14 | BOLD |
| *Aphis fabae* | ACEA086-14 | BOLD |
| *Aphis fabae* | ACEA087-14 | BOLD |
| *Aphis rumicis* | ACEA088-14 | BOLD |
| *Aphis fabae* | ACEA089-14 | BOLD |
| *Aphis fabae* | ACEA090-14 | BOLD |
| *Aphis fabae* | ACEA091-14 | BOLD |
| *Aphis fabae* | ACEA092-14 | BOLD |
| *Aphis fabae* | ACEA093-14 | BOLD |
| *Aphis viburni* | ACEA094-14 | BOLD |
| *Aphis fabae* | ACEA095-14 | BOLD |
| *Aphis fabae* | ACEA096-14 | BOLD |
| *Aphis fabae* | ACEA097-14 | BOLD |
| *Aphis fabae* | ACEA098-14 | BOLD |
| *Aphis fabae* | ACEA099-14 | BOLD |
| *Aphis veratri* | ACEA100-14 | BOLD |
| *Brachycaudus helichrysi* | ACEA1000-14 | BOLD |
| *Aphis caroliboerneri* | ACEA1001-14 | BOLD |
| *Therioaphis riehmi* | ACEA1002-14 | BOLD |
| *Myzus lythri* | ACEA1003-14 | BOLD |
| *Aphis clematidis* | ACEA1004-14 | BOLD |
| *Aphis oenotherae* | ACEA1005-14 | BOLD |
| *Brachycaudus helichrysi* | ACEA1006-14 | BOLD |
| *Brachycaudus helichrysi* | ACEA1007-14 | BOLD |
| *Aphis cisticola* | ACEA1008-14 | BOLD |
| *Hyperomyzus rhinanthi* | ACEA1009-14 | BOLD |
| *Aphis fabae* | ACEA101-14 | BOLD |
| *Brachycaudus populi* | ACEA1010-14 | BOLD |
| *Pterocomma rufipes* | ACEA1011-14 | BOLD |
| *Cinara cuneomaculata* | ACEA1012-14 | BOLD |
| *Aphis farinosa* | ACEA1013-14 | BOLD |
| *Pterocomma pilosum* | ACEA1014-14 | BOLD |
| *Aphis spiraecola* | ACEA1015-14 | BOLD |
| *Aphis spiraecola* | ACEA1016-14 | BOLD |
| *Aphis pomi* | ACEA1017-14 | BOLD |
| *Aphis pomi* | ACEA1018-14 | BOLD |
| *Aphis pomi* | ACEA1019-14 | BOLD |
| *Aphis spiraecola* | ACEA1020-14 | BOLD |
| *Periphyllus testudinaceus* | ACEA103-14 | BOLD |
| *Ovatus crataegarius* | ACEA104-14 | BOLD |
| *Aphis clematidis* | ACEA105-14 | BOLD |
| *Macrosiphoniella helichrysi* | ACEA106-14 | BOLD |
| *Uroleucon jaceae* | ACEA107-14 | BOLD |
| *Ephedraphis ephedrae* | ACEA108-14 | BOLD |
| *Melanaphis donacis* | ACEA109-14 | BOLD |
| *Anoecia corni* | ACEA110-14 | BOLD |
| *Aphis sanguisorbae* | ACEA111-14 | BOLD |
| *Aphis sanguisorbae* | ACEA112-14 | BOLD |
| *Aphis fabae* | ACEA113-14 | BOLD |
| *Chaitophorus leucomelas* | ACEA114-14 | BOLD |
| *Pterocomma populeum* | ACEA115-14 | BOLD |
| *Acyrthosiphon pisum* | ACEA116-14 | BOLD |
| *Brachycaudus tragopogonis* | ACEA118-14 | BOLD |
| *Protaphis terricola* | ACEA119-14 | BOLD |
| *Protaphis terricola* | ACEA120-14 | BOLD |
| *Uroleucon jaceae* | ACEA121-14 | BOLD |
| *Brachyunguis tamaricis* | ACEA122-14 | BOLD |
| *Periphyllus bulgaricus* | ACEA123-14 | BOLD |
| *Myzus lythri* | ACEA124-14 | BOLD |
| *Aphis fabae* | ACEA125-14 | BOLD |
| *Dysaphis radicola* | ACEA126-14 | BOLD |
| *Hyperomyzus lactucae* | ACEA127-14 | BOLD |
| *Uroleucon sonchi* | ACEA128-14 | BOLD |
| *Aphis fabae* | ACEA129-14 | BOLD |
| *Aphis spiraecola* | ACEA130-14 | BOLD |
| *Aulacorthum solani* | ACEA131-14 | BOLD |
| *Microlophium carnosum* | ACEA132-14 | BOLD |
| *Aphis ruborum* | ACEA133-14 | BOLD |
| *Aphis fabae* | ACEA134-14 | BOLD |
| *Macrosiphum rosae* | ACEA135-14 | BOLD |
| *Megourella tribulis* | ACEA136-14 | BOLD |
| *Macrosiphoniella oblonga* | ACEA137-14 | BOLD |
| *Dysaphis aucupariae* | ACEA139-14 | BOLD |
| *Aphis fabae* | ACEA140-14 | BOLD |
| *Aphis craccivora* | ACEA141-14 | BOLD |
| *Uroleucon jaceae* | ACEA142-14 | BOLD |
| *Aphis gossypii* | ACEA143-14 | BOLD |
| *Uroleucon nigrocampanulae* | ACEA144-14 | BOLD |
| *Aphis aliena* | ACEA145-14 | BOLD |
| *Aphis taraxacicola* | ACEA146-14 | BOLD |
| *Uroleucon hypochoeridis* | ACEA147-14 | BOLD |
| *Aphis urticata* | ACEA148-14 | BOLD |
| *Macrosiphum rosae* | ACEA149-14 | BOLD |
| *Brachycaudus tragopogonis* | ACEA150-14 | BOLD |
| *Brachycaudus cardui* | ACEA151-14 | BOLD |
| *Uroleucon hypochoeridis* | ACEA152-14 | BOLD |
| *Pterocomma pilosum* | ACEA153-14 | BOLD |
| *Cinara pilicornis* | ACEA154-14 | BOLD |
| *Aphis taraxacicola* | ACEA155-14 | BOLD |
| *Hyalopterus pruni* | ACEA156-14 | BOLD |
| *Aphis ruborum* | ACEA157-14 | BOLD |
| *Brachycaudus cardui* | ACEA158-14 | BOLD |
| *Myzus lythri* | ACEA159-14 | BOLD |
| *Cinara juniperi* | ACEA160-14 | BOLD |
| *Aphis salviae* | ACEA161-14 | BOLD |
| *Dysaphis apiifolia* | ACEA162-14 | BOLD |
| *Brachycaudus lychnidis* | ACEA163-14 | BOLD |
| *Anuraphis subterranea* | ACEA164-14 | BOLD |
| *Hyalopterus pruni* | ACEA165-14 | BOLD |
| *Pleotrichophorus glandulosus* | ACEA166-14 | BOLD |
| *Brachycaudus lychnidis* | ACEA167-14 | BOLD |
| *Uroleucon hypochoeridis* | ACEA168-14 | BOLD |
| *Thecabius affinis* | ACEA169-14 | BOLD |
| *Pemphigus vesicarius* | ACEA170-14 | BOLD |
| *Pemphigus immunis* | ACEA171-14 | BOLD |
| *Aphis craccivora* | ACEA172-14 | BOLD |
| *Aphis fabae* | ACEA173-14 | BOLD |
| *Cinara pruinosa* | ACEA174-14 | BOLD |
| *Aphis fabae* | ACEA175-14 | BOLD |
| *Aphis umbrella* | ACEA176-14 | BOLD |
| *Aphis sambuci* | ACEA177-14 | BOLD |
| *Aphis crepidis* | ACEA178-14 | BOLD |
| *Aphis gossypii* | ACEA179-14 | BOLD |
| *Aphis tirucallis* | ACEA181-14 | BOLD |
| *Pterocomma populeum* | ACEA182-14 | BOLD |
| *Panaphis juglandis* | ACEA183-14 | BOLD |
| *Aphis spiraecola* | ACEA184-14 | BOLD |
| *Aphis newtoni* | ACEA185-14 | BOLD |
| *Brachycaudus schwartzi* | ACEA186-14 | BOLD |
| *Brachycaudus cardui* | ACEA187-14 | BOLD |
| *Aphis verbasci* | ACEA188-14 | BOLD |
| *Aphis fabae* | ACEA189-14 | BOLD |
| *Aphis fabae* | ACEA190-14 | BOLD |
| *Aphis pseudocomosa* | ACEA191-14 | BOLD |
| *Uroleucon achilleae* | ACEA192-14 | BOLD |
| *Toxopterina vandergooti* | ACEA193-14 | BOLD |
| *Macrosiphoniella artemisiae* | ACEA195-14 | BOLD |
| *Periphyllus testudinaceus* | ACEA196-14 | BOLD |
| *Aphis cacaliasteris* | ACEA197-14 | BOLD |
| *Macrosiphum rosae* | ACEA198-14 | BOLD |
| *Aphis grossulariae* | ACEA199-14 | BOLD |
| *Aphis fabae* | ACEA200-14 | BOLD |
| *Aphis confusa* | ACEA201-14 | BOLD |
| *Toxopterina vandergooti* | ACEA202-14 | BOLD |
| *Aphis fabae* | ACEA203-14 | BOLD |
| *Aphis fabae* | ACEA204-14 | BOLD |
| *Brachycaudus helichrysi* | ACEA205-14 | BOLD |
| *Aphis fabae* | ACEA206-14 | BOLD |
| *Brachycaudus helichrysi* | ACEA207-14 | BOLD |
| *Aphis fabae* | ACEA208-14 | BOLD |
| *Aphis leontodontis* | ACEA209-14 | BOLD |
| *Aphis sambuci* | ACEA210-14 | BOLD |
| *Aphis pomi* | ACEA211-14 | BOLD |
| *Macrosiphum rosae* | ACEA212-14 | BOLD |
| *Aphis pomi* | ACEA213-14 | BOLD |
| *Uroleucon sonchi* | ACEA214-14 | BOLD |
| *Uroleucon hypochoeridis* | ACEA215-14 | BOLD |
| *Aphis grossulariae* | ACEA216-14 | BOLD |
| *Aphis ruborum* | ACEA217-14 | BOLD |
| *Aphis coronillae* | ACEA218-14 | BOLD |
| *Aphis fabae* | ACEA219-14 | BOLD |
| *Brachycaudus lateralis* | ACEA220-14 | BOLD |
| *Hyperomyzus lactucae* | ACEA221-14 | BOLD |
| *Semiaphis dauci* | ACEA222-14 | BOLD |
| *Dysaphis crataegi* | ACEA223-14 | BOLD |
| *Aphis coronillae* | ACEA224-14 | BOLD |
| *Uroleucon hypochoeridis* | ACEA225-14 | BOLD |
| *Dysaphis radicola* | ACEA226-14 | BOLD |
| *Myzocallis coryli* | ACEA227-14 | BOLD |
| *Aphis fabae* | ACEA228-14 | BOLD |
| *Brachycaudus lateralis* | ACEA229-14 | BOLD |
| *Hyalopterus pruni* | ACEA230-14 | BOLD |
| *Aphis fabae* | ACEA231-14 | BOLD |
| *Aphis newtoni* | ACEA232-14 | BOLD |
| *Macrosiphum rosae* | ACEA233-14 | BOLD |
| *Thelaxes dryophila* | ACEA234-14 | BOLD |
| *Pterocallis alni* | ACEA235-14 | BOLD |
| *Cinara pilicornis* | ACEA236-14 | BOLD |
| *Aphis fabae* | ACEA237-14 | BOLD |
| *Brachycaudus helichrysi* | ACEA238-14 | BOLD |
| *Corylobium avellanae* | ACEA239-14 | BOLD |
| *Stomaphis longirostris* | ACEA240-14 | BOLD |
| *Cinara maghrebica* | ACEA241-14 | BOLD |
| *Nearctaphis bakeri* | ACEA242-14 | BOLD |
| *Aphis serpylli* | ACEA243-14 | BOLD |
| *Macchiatiella rhamni* | ACEA244-14 | BOLD |
| *Aphis cytisorum* | ACEA245-14 | BOLD |
| *Hyperomyzus picridis* | ACEA246-14 | BOLD |
| *Sitobion fragariae* | ACEA247-14 | BOLD |
| *Dysaphis crataegi* | ACEA249-14 | BOLD |
| *Macrosiphum euphorbiae* | ACEA250-14 | BOLD |
| *Aphis oenotherae* | ACEA251-14 | BOLD |
| *Chaitophorus truncatus* | ACEA252-14 | BOLD |
| *Aphis fabae* | ACEA253-14 | BOLD |
| *Aphis cytisorum* | ACEA254-14 | BOLD |
| *Chaitophorus leucomelas* | ACEA255-14 | BOLD |
| *Aphis lambersi* | ACEA256-14 | BOLD |
| *Uroleucon hypochoeridis* | ACEA257-14 | BOLD |
| *Aphis craccivora* | ACEA258-14 | BOLD |
| *Aphis fabae* | ACEA259-14 | BOLD |
| *Chaitophorus populeti* | ACEA260-14 | BOLD |
| *Hyperomyzus lactucae* | ACEA261-14 | BOLD |
| *Aphis spiraecola* | ACEA262-14 | BOLD |
| *Aphis fabae* | ACEA263-14 | BOLD |
| *Macrosiphum euphorbiae* | ACEA264-14 | BOLD |
| *Melanaphis donacis* | ACEA265-14 | BOLD |
| *Brachycaudus lychnidis* | ACEA266-14 | BOLD |
| *Uroleucon jaceae* | ACEA267-14 | BOLD |
| *Chaitophorus leucomelas* | ACEA268-14 | BOLD |
| *Myzus cerasi* | ACEA269-14 | BOLD |
| *Hyperomyzus lactucae* | ACEA270-14 | BOLD |
| *Aphis lichtensteini* | ACEA271-14 | BOLD |
| *Dysaphis apiifolia* | ACEA272-14 | BOLD |
| *Uroleucon hypochoeridis* | ACEA273-14 | BOLD |
| *Aphis oenotherae* | ACEA274-14 | BOLD |
| *Aphis sedi* | ACEA275-14 | BOLD |
| *Macrosiphum euphorbiae* | ACEA276-14 | BOLD |
| *Hyalopterus pruni* | ACEA277-14 | BOLD |
| *Therioaphis riehmi* | ACEA278-14 | BOLD |
| *Brachycaudus lateralis* | ACEA279-14 | BOLD |
| *Uroleucon sonchi* | ACEA280-14 | BOLD |
| *Dysaphis apiifolia* | ACEA281-14 | BOLD |
| *Chaitophorus populeti* | ACEA282-14 | BOLD |
| *Aphis fabae* | ACEA283-14 | BOLD |
| *Aphis fabae* | ACEA284-14 | BOLD |
| *Chaitophorus populeti* | ACEA285-14 | BOLD |
| *Chaitophorus populialbae* | ACEA286-14 | BOLD |
| *Aphis oenotherae* | ACEA287-14 | BOLD |
| *Aphis ulicis* | ACEA288-14 | BOLD |
| *Aphis epilobii* | ACEA289-14 | BOLD |
| *Amphorophora rubi* | ACEA290-14 | BOLD |
| *Brachycaudus bicolor* | ACEA291-14 | BOLD |
| *Aphis longirostrata* | ACEA292-14 | BOLD |
| *Hyperomyzus lactucae* | ACEA293-14 | BOLD |
| *Uroleucon hypochoeridis* | ACEA294-14 | BOLD |
| *Hayhurstia atriplicis* | ACEA295-14 | BOLD |
| *Brachycaudus lateralis* | ACEA296-14 | BOLD |
| *Dysaphis crithmi* | ACEA297-14 | BOLD |
| *Cavariella theobaldi* | ACEA298-14 | BOLD |
| *Hyperomyzus lactucae* | ACEA299-14 | BOLD |
| *Hayhurstia atriplicis* | ACEA300-14 | BOLD |
| *Aphis fabae* | ACEA301-14 | BOLD |
| *Chaitophorus leucomelas* | ACEA302-14 | BOLD |
| *Dysaphis plantaginea* | ACEA303-14 | BOLD |
| *Aphis ruborum* | ACEA304-14 | BOLD |
| *Aphis sanguisorbae* | ACEA305-14 | BOLD |
| *Aphis fabae* | ACEA306-14 | BOLD |
| *Brachycaudus prunicola* | ACEA307-14 | BOLD |
| *Aphis ruborum* | ACEA308-14 | BOLD |
| *Tuberculatus annulatus* | ACEA309-14 | BOLD |
| *Aphis newtoni* | ACEA310-14 | BOLD |
| *Aphis fabae* | ACEA311-14 | BOLD |
| *Aphis rumicis* | ACEA312-14 | BOLD |
| *Uroleucon hypochoeridis* | ACEA313-14 | BOLD |
| *Aphis grossulariae* | ACEA314-14 | BOLD |
| *Thelaxes dryophila* | ACEA315-14 | BOLD |
| *Aphis acetosae* | ACEA316-14 | BOLD |
| *Hyalopterus pruni* | ACEA317-14 | BOLD |
| *Aphis craccivora* | ACEA318-14 | BOLD |
| *Chaitophorus salicti* | ACEA319-14 | BOLD |
| *Aphis serpylli* | ACEA320-14 | BOLD |
| *Hydaphias mosana* | ACEA321-14 | BOLD |
| *Cavariella theobaldi* | ACEA322-14 | BOLD |
| *Dysaphis lauberti* | ACEA324-14 | BOLD |
| *Hyalopterus pruni* | ACEA325-14 | BOLD |
| *Uroleucon picridis* | ACEA326-14 | BOLD |
| *Dysaphis crithmi* | ACEA327-14 | BOLD |
| *Aphis lambersi* | ACEA328-14 | BOLD |
| *Dysaphis crataegi* | ACEA329-14 | BOLD |
| *Uroleucon jaceae* | ACEA330-14 | BOLD |
| *Nearctaphis bakeri* | ACEA331-14 | BOLD |
| *Aphis ulicis* | ACEA332-14 | BOLD |
| *Aphis fabae* | ACEA333-14 | BOLD |
| *Macrosiphum euphorbiae* | ACEA334-14 | BOLD |
| *Brachycaudus klugkisti* | ACEA335-14 | BOLD |
| *Uroleucon hypochoeridis* | ACEA336-14 | BOLD |
| *Amphorophora rubi* | ACEA337-14 | BOLD |
| *Aphis ilicis* | ACEA338-14 | BOLD |
| *Aphis fabae* | ACEA339-14 | BOLD |
| *Aphis sedi* | ACEA340-14 | BOLD |
| *Brevicoryne brassicae* | ACEA341-14 | BOLD |
| *Aphis nerii* | ACEA342-14 | BOLD |
| *Cinara palaestinensis* | ACEA343-14 | BOLD |
| *Protaphis terricola* | ACEA344-14 | BOLD |
| *Aphis craccivora* | ACEA345-14 | BOLD |
| *Aphis gossypii* | ACEA346-14 | BOLD |
| *Aphis craccivora* | ACEA347-14 | BOLD |
| *Aphis craccivora* | ACEA348-14 | BOLD |
| *Uroleucon inulae* | ACEA349-14 | BOLD |
| *Aphis craccivora* | ACEA351-14 | BOLD |
| *Acyrthosiphon lambersi* | ACEA352-14 | BOLD |
| *Anuraphis cachryos* | ACEA353-14 | BOLD |
| *Chaitophorus populeti* | ACEA354-14 | BOLD |
| *Protaphis terricola* | ACEA355-14 | BOLD |
| *Chaitophorus populeti* | ACEA356-14 | BOLD |
| *Chaitophorus leucomelas* | ACEA357-14 | BOLD |
| *Pterocomma populeum* | ACEA358-14 | BOLD |
| *Aphis craccivora* | ACEA359-14 | BOLD |
| *Chaitophorus populeti* | ACEA360-14 | BOLD |
| *Aphis spiraecola* | ACEA361-14 | BOLD |
| *Brevicoryne brassicae* | ACEA362-14 | BOLD |
| *Periphyllus acericola* | ACEA363-14 | BOLD |
| *Hyperomyzus lactucae* | ACEA364-14 | BOLD |
| *Aphis hederae* | ACEA365-14 | BOLD |
| *Brachycaudus persicae* | ACEA366-14 | BOLD |
| *Anuraphis subterranea* | ACEA367-14 | BOLD |
| *Macrosiphum euphorbiae* | ACEA368-14 | BOLD |
| *Aphis urticata* | ACEA369-14 | BOLD |
| *Hyperomyzus lactucae* | ACEA370-14 | BOLD |
| *Aphis fabae* | ACEA371-14 | BOLD |
| *Aphis lugentis* | ACEA372-14 | BOLD |
| *Aphis fabae* | ACEA373-14 | BOLD |
| *Megoura viciae* | ACEA374-14 | BOLD |
| *Sipha elegans* | ACEA375-14 | BOLD |
| *Macrosiphum rosae* | ACEA376-14 | BOLD |
| *Brachycaudus lychnidis* | ACEA377-14 | BOLD |
| *Aphis umbrella* | ACEA378-14 | BOLD |
| *Semiaphis dauci* | ACEA379-14 | BOLD |
| *Lachnus roboris* | ACEA380-14 | BOLD |
| *Aphis craccivora* | ACEA381-14 | BOLD |
| *Aphis fabae* | ACEA382-14 | BOLD |
| *Cinara pinea* | ACEA383-14 | BOLD |
| *Nearctaphis bakeri* | ACEA384-14 | BOLD |
| *Macrosiphum rosae* | ACEA385-14 | BOLD |
| *Periphyllus testudinaceus* | ACEA386-14 | BOLD |
| *Hyperomyzus picridis* | ACEA387-14 | BOLD |
| *Myzus cerasi* | ACEA388-14 | BOLD |
| *Prociphilus bumeliae* | ACEA389-14 | BOLD |
| *Brachycaudus klugkisti* | ACEA390-14 | BOLD |
| *Aphis lugentis* | ACEA391-14 | BOLD |
| *Aphis fabae* | ACEA392-14 | BOLD |
| *Macrosiphum euphorbiae* | ACEA393-14 | BOLD |
| *Prociphilus bumeliae* | ACEA394-14 | BOLD |
| *Aphis fabae* | ACEA395-14 | BOLD |
| *Amphorophora rubi* | ACEA396-14 | BOLD |
| *Macrosiphum euphorbiae* | ACEA397-14 | BOLD |
| *Aphis urticata* | ACEA398-14 | BOLD |
| *Chaitophorus populeti* | ACEA399-14 | BOLD |
| *Aphis confusa* | ACEA400-14 | BOLD |
| *Forda marginata* | ACEA401-14 | BOLD |
| *Hyadaphis foeniculi* | ACEA402-14 | BOLD |
| *Pterocomma pilosum* | ACEA403-14 | BOLD |
| *Macrosiphoniella millefolii* | ACEA404-14 | BOLD |
| *Brachycaudus helichrysi* | ACEA405-14 | BOLD |
| *Aphis sambuci* | ACEA406-14 | BOLD |
| *Aphis viburni* | ACEA407-14 | BOLD |
| *Aphis farinosa* | ACEA408-14 | BOLD |
| *Myzocallis coryli* | ACEA409-14 | BOLD |
| *Chaitophorus populeti* | ACEA410-14 | BOLD |
| *Chaitophorus salicti* | ACEA411-14 | BOLD |
| *Periphyllus testudinaceus* | ACEA412-14 | BOLD |
| *Aphis grossulariae* | ACEA413-14 | BOLD |
| *Chaitophorus capreae* | ACEA414-14 | BOLD |
| *Myzocallis carpini* | ACEA415-14 | BOLD |
| *Aphis ilicis* | ACEA416-14 | BOLD |
| *Macrosiphum rosae* | ACEA417-14 | BOLD |
| *Myzus cerasi* | ACEA418-14 | BOLD |
| *Macrosiphoniella tanacetaria* | ACEA419-14 | BOLD |
| *Cavariella theobaldi* | ACEA420-14 | BOLD |
| *Macrosiphum rosae* | ACEA421-14 | BOLD |
| *Aphis fabae* | ACEA422-14 | BOLD |
| *Macrosiphum rosae* | ACEA423-14 | BOLD |
| *Brachycaudus tragopogonis* | ACEA425-14 | BOLD |
| *Brachycaudus cardui* | ACEA426-14 | BOLD |
| *Phorodon humuli* | ACEA427-14 | BOLD |
| *Aphis oenotherae* | ACEA428-14 | BOLD |
| *Brachycaudus cardui* | ACEA429-14 | BOLD |
| *Brachycaudus lychnidis* | ACEA430-14 | BOLD |
| *Aphis urticata* | ACEA431-14 | BOLD |
| *Dysaphis lauberti* | ACEA432-14 | BOLD |
| *Aphis ruborum* | ACEA433-14 | BOLD |
| *Amphorophora rubi* | ACEA434-14 | BOLD |
| *Dysaphis crithmi* | ACEA435-14 | BOLD |
| *Macrosiphum euphorbiae* | ACEA436-14 | BOLD |
| *Tuberculatus annulatus* | ACEA437-14 | BOLD |
| *Thelaxes dryophila* | ACEA438-14 | BOLD |
| *Brevicoryne brassicae* | ACEA439-14 | BOLD |
| *Phyllaphis fagi* | ACEA440-14 | BOLD |
| *Phorodon humuli* | ACEA441-14 | BOLD |
| *Brevicoryne brassicae* | ACEA442-14 | BOLD |
| *Brachycaudus cardui* | ACEA443-14 | BOLD |
| *Trama troglodytes* | ACEA444-14 | BOLD |
| *Aphis gossypii* | ACEA445-14 | BOLD |
| *Uroleucon jaceae* | ACEA446-14 | BOLD |
| *Periphyllus testudinaceus* | ACEA447-14 | BOLD |
| *Brachycaudus cardui* | ACEA448-14 | BOLD |
| *Microlophium carnosum* | ACEA449-14 | BOLD |
| *Betulaphis brevipilosa* | ACEA450-14 | BOLD |
| *Dysaphis radicola* | ACEA451-14 | BOLD |
| *Hyperomyzus picridis* | ACEA452-14 | BOLD |
| *Aphis fabae* | ACEA453-14 | BOLD |
| *Macrosiphum euphorbiae* | ACEA454-14 | BOLD |
| *Euceraphis punctipennis* | ACEA455-14 | BOLD |
| *Aphis oenotherae* | ACEA456-14 | BOLD |
| *Cavariella theobaldi* | ACEA457-14 | BOLD |
| *Macrosiphum euphorbiae* | ACEA458-14 | BOLD |
| *Eucallipterus tiliae* | ACEA459-14 | BOLD |
| *Pterocallis alni* | ACEA460-14 | BOLD |
| *Aphis taraxacicola* | ACEA461-14 | BOLD |
| *Cinara pinea* | ACEA462-14 | BOLD |
| *Aphis fabae* | ACEA463-14 | BOLD |
| *Thelaxes dryophila* | ACEA464-14 | BOLD |
| *Myzus cerasi* | ACEA465-14 | BOLD |
| *Hyalopterus pruni* | ACEA466-14 | BOLD |
| *Brachycaudus cardui* | ACEA467-14 | BOLD |
| *Aphis coronillae* | ACEA468-14 | BOLD |
| *Aphis pomi* | ACEA469-14 | BOLD |
| *Myzocallis coryli* | ACEA470-14 | BOLD |
| *Macrosiphum euphorbiae* | ACEA471-14 | BOLD |
| *Euceraphis betulae* | ACEA472-14 | BOLD |
| *Hyalopterus pruni* | ACEA473-14 | BOLD |
| *Aphis pomi* | ACEA474-14 | BOLD |
| *Tuberculatus annulatus* | ACEA475-14 | BOLD |
| *Thelaxes dryophila* | ACEA476-14 | BOLD |
| *Cinara pinimaritimae* | ACEA477-14 | BOLD |
| *Aphis ulmariae* | ACEA478-14 | BOLD |
| *Aphis cytisorum* | ACEA479-14 | BOLD |
| *Dysaphis angelicae* | ACEA480-14 | BOLD |
| *Aphis hederae* | ACEA481-14 | BOLD |
| *Aphis spiraecola* | ACEA482-14 | BOLD |
| *Aphis fabae* | ACEA483-14 | BOLD |
| *Aphis fabae* | ACEA484-14 | BOLD |
| *Aphis fabae* | ACEA485-14 | BOLD |
| *Tuberculatus annulatus* | ACEA486-14 | BOLD |
| *Nearctaphis bakeri* | ACEA487-14 | BOLD |
| *Aphis pomi* | ACEA488-14 | BOLD |
| *Aphis epilobii* | ACEA489-14 | BOLD |
| *Aphis sambuci* | ACEA490-14 | BOLD |
| *Aphis grossulariae* | ACEA491-14 | BOLD |
| *Chaitophorus populeti* | ACEA492-14 | BOLD |
| *Uroleucon hypochoeridis* | ACEA493-14 | BOLD |
| *Aphis fabae* | ACEA494-14 | BOLD |
| *Myzocallis castanicola* | ACEA495-14 | BOLD |
| *Cavariella theobaldi* | ACEA496-14 | BOLD |
| *Aphis jacobaeae* | ACEA497-14 | BOLD |
| *Aphis ulicis* | ACEA498-14 | BOLD |
| *Uroleucon sonchi* | ACEA499-14 | BOLD |
| *Uroleucon sonchi* | ACEA500-14 | BOLD |
| *Macrosiphoniella artemisiae* | ACEA501-14 | BOLD |
| *Lachnus roboris* | ACEA502-14 | BOLD |
| *Illinoia liriodendri* | ACEA503-14 | BOLD |
| *Aphis grossulariae* | ACEA504-14 | BOLD |
| *Aphis hederae* | ACEA505-14 | BOLD |
| *Aphis spiraecola* | ACEA506-14 | BOLD |
| *Uroleucon sonchi* | ACEA507-14 | BOLD |
| *Chaitophorus leucomelas* | ACEA508-14 | BOLD |
| *Aphis pomi* | ACEA509-14 | BOLD |
| *Chaitophorus populialbae* | ACEA510-14 | BOLD |
| *Aphis newtoni* | ACEA511-14 | BOLD |
| *Aphis oenotherae* | ACEA512-14 | BOLD |
| *Uroleucon sonchi* | ACEA513-14 | BOLD |
| *Brachycaudus bicolor* | ACEA514-14 | BOLD |
| *Chaitophorus populialbae* | ACEA515-14 | BOLD |
| *Aulacorthum solani* | ACEA516-14 | BOLD |
| *Lipaphis lepidii* | ACEA517-14 | BOLD |
| *Uroleucon sonchi* | ACEA518-14 | BOLD |
| *Periphyllus testudinaceus* | ACEA519-14 | BOLD |
| *Dysaphis lappae* | ACEA520-14 | BOLD |
| *Brachycaudus cardui* | ACEA521-14 | BOLD |
| *Macrosiphum euphorbiae* | ACEA522-14 | BOLD |
| *Hyadaphis passerinii* | ACEA523-14 | BOLD |
| *Aphis fabae* | ACEA524-14 | BOLD |
| *Euceraphis betulae* | ACEA525-14 | BOLD |
| *Aphis grossulariae* | ACEA526-14 | BOLD |
| *Aphis intybi* | ACEA527-14 | BOLD |
| *Aphis viticis* | ACEA528-14 | BOLD |
| *Uroleucon sonchi* | ACEA529-14 | BOLD |
| *Aphis nasturtii* | ACEA530-14 | BOLD |
| *Aphis fabae* | ACEA531-14 | BOLD |
| *Rhopalosiphum padi* | ACEA532-14 | BOLD |
| *Dysaphis crataegi* | ACEA534-14 | BOLD |
| *Brevicoryne brassicae* | ACEA535-14 | BOLD |
| *Aphis craccivora* | ACEA536-14 | BOLD |
| *Aphis serpylli* | ACEA537-14 | BOLD |
| *Uroleucon jaceae* | ACEA538-14 | BOLD |
| *Uroleucon hypochoeridis* | ACEA539-14 | BOLD |
| *Aphis intybi* | ACEA540-14 | BOLD |
| *Uroleucon aeneum* | ACEA541-14 | BOLD |
| *Uroleucon hypochoeridis* | ACEA542-14 | BOLD |
| *Brachyunguis tamaricis* | ACEA543-14 | BOLD |
| *Hyalopterus pruni* | ACEA544-14 | BOLD |
| *Uroleucon sonchi* | ACEA545-14 | BOLD |
| *Therioaphis riehmi* | ACEA546-14 | BOLD |
| *Dysaphis crataegi* | ACEA547-14 | BOLD |
| *Aphis fabae* | ACEA548-14 | BOLD |
| *Chaitophorus salijaponicus* | ACEA549-14 | BOLD |
| *Aphis ruborum* | ACEA550-14 | BOLD |
| *Aphis gossypii* | ACEA551-14 | BOLD |
| *Aphis craccivora* | ACEA552-14 | BOLD |
| *Hyperomyzus lactucae* | ACEA554-14 | BOLD |
| *Aphis gossypii* | ACEA555-14 | BOLD |
| *Aphis gossypii* | ACEA556-14 | BOLD |
| *Aphis craccivora* | ACEA557-14 | BOLD |
| *Dysaphis apiifolia* | ACEA558-14 | BOLD |
| *Hyperomyzus lactucae* | ACEA559-14 | BOLD |
| *Hyalopterus persikonus* | ACEA560-14 | BOLD |
| *Aphis hederae* | ACEA561-14 | BOLD |
| *Macrosiphum rosae* | ACEA562-14 | BOLD |
| *Aphis gossypii* | ACEA563-14 | BOLD |
| *Aphis gossypii* | ACEA564-14 | BOLD |
| *Aphis craccivora* | ACEA565-14 | BOLD |
| *Hayhurstia atriplicis* | ACEA566-14 | BOLD |
| *Aphis gossypii* | ACEA567-14 | BOLD |
| *Aphis gossypii* | ACEA568-14 | BOLD |
| *Aphis ruborum* | ACEA569-14 | BOLD |
| *Aphis gossypii* | ACEA570-14 | BOLD |
| *Aphis craccivora* | ACEA571-14 | BOLD |
| *Macrosiphum rosae* | ACEA572-14 | BOLD |
| *Melanaphis pyraria* | ACEA573-14 | BOLD |
| *Aphis brotericola* | ACEA574-14 | BOLD |
| *Aphis fabae* | ACEA575-14 | BOLD |
| *Aphis craccae* | ACEA576-14 | BOLD |
| *Thelaxes suberi* | ACEA577-14 | BOLD |
| *Brachycaudus tragopogonis* | ACEA578-14 | BOLD |
| *Aphis fabae* | ACEA579-14 | BOLD |
| *Brachycaudus helichrysi* | ACEA580-14 | BOLD |
| *Aphis gossypii* | ACEA581-14 | BOLD |
| *Aphis vitalbae* | ACEA582-14 | BOLD |
| *Brachycaudus bicolor* | ACEA583-14 | BOLD |
| *Dysaphis reaumuri* | ACEA584-14 | BOLD |
| *Dysaphis crataegi* | ACEA585-14 | BOLD |
| *Aphis cisticola* | ACEA586-14 | BOLD |
| *Aphis origani* | ACEA587-14 | BOLD |
| *Dysaphis crataegi* | ACEA588-14 | BOLD |
| *Aphis spiraecola* | ACEA589-14 | BOLD |
| *Aphis spiraecola* | ACEA590-14 | BOLD |
| *Panaphis juglandis* | ACEA591-14 | BOLD |
| *Brachycaudus populi* | ACEA593-14 | BOLD |
| *Aphis vallei* | ACEA594-14 | BOLD |
| *Lachnus roboris* | ACEA595-14 | BOLD |
| *Acyrthosiphon caraganae* | ACEA597-14 | BOLD |
| *Aphis punicae* | ACEA599-14 | BOLD |
| *Aphis spiraecola* | ACEA600-14 | BOLD |
| *Aphis chloris* | ACEA601-14 | BOLD |
| *Sitobion fragariae* | ACEA602-14 | BOLD |
| *Chaitophorus nigricantis* | ACEA603-14 | BOLD |
| *Melanaphis donacis* | ACEA604-14 | BOLD |
| *Chaitophorus leucomelas* | ACEA605-14 | BOLD |
| *Aphis ruborum* | ACEA606-14 | BOLD |
| *Aphis viticis* | ACEA607-14 | BOLD |
| *Aphis fabae* | ACEA608-14 | BOLD |
| *Uroleucon rapunculoidis* | ACEA609-14 | BOLD |
| *Aphis craccae* | ACEA610-14 | BOLD |
| *Myzocallis castanicola* | ACEA611-14 | BOLD |
| *Aphis cytisorum* | ACEA612-14 | BOLD |
| *Aphis vitalbae* | ACEA613-14 | BOLD |
| *Chaitophorus populialbae* | ACEA614-14 | BOLD |
| *Aphis craccivora* | ACEA615-14 | BOLD |
| *Chaitophorus populeti* | ACEA616-14 | BOLD |
| *Thelaxes suberi* | ACEA618-14 | BOLD |
| *Aphis origani* | ACEA619-14 | BOLD |
| *Aphis cytisorum* | ACEA621-14 | BOLD |
| *Aphis fabae* | ACEA622-14 | BOLD |
| *Uroleucon sonchi* | ACEA624-14 | BOLD |
| *Aphis craccivora* | ACEA625-14 | BOLD |
| *Chaitophorus leucomelas* | ACEA626-14 | BOLD |
| *Aphis nerii* | ACEA627-14 | BOLD |
| *Uroleucon hypochoeridis* | ACEA628-14 | BOLD |
| *Aphis craccivora* | ACEA629-14 | BOLD |
| *Aphis spiraecola* | ACEA630-14 | BOLD |
| *Macrosiphum euphorbiae* | ACEA631-14 | BOLD |
| *Lipaphis erysimi* | ACEA632-14 | BOLD |
| *Cinara palaestinensis* | ACEA633-14 | BOLD |
| *Aphis craccivora* | ACEA634-14 | BOLD |
| *Aphis spiraecola* | ACEA635-14 | BOLD |
| *Chaitophorus leucomelas* | ACEA636-14 | BOLD |
| *Pterocomma populeum* | ACEA637-14 | BOLD |
| *Uroleucon hypochoeridis* | ACEA638-14 | BOLD |
| *Aphis hypochoeridis* | ACEA639-14 | BOLD |
| *Aphis craccivora* | ACEA640-14 | BOLD |
| *Aphis sanguisorbae* | ACEA641-14 | BOLD |
| *Aphis fabae* | ACEA642-14 | BOLD |
| *Thelaxes suberi* | ACEA643-14 | BOLD |
| *Semiaphis dauci* | ACEA644-14 | BOLD |
| *Aphis ruborum* | ACEA645-14 | BOLD |
| *Sitobion fragariae* | ACEA646-14 | BOLD |
| *Aphis fabae* | ACEA647-14 | BOLD |
| *Uroleucon hypochoeridis* | ACEA648-14 | BOLD |
| *Chaitophorus salijaponicus* | ACEA649-14 | BOLD |
| *Chaitophorus salijaponicus* | ACEA650-14 | BOLD |
| *Chaitophorus salijaponicus* | ACEA651-14 | BOLD |
| *Microlophium carnosum* | ACEA652-14 | BOLD |
| *Aphis serpylli* | ACEA653-14 | BOLD |
| *Aphis fabae* | ACEA654-14 | BOLD |
| *Aphis urticata* | ACEA655-14 | BOLD |
| *Aphis sambuci* | ACEA656-14 | BOLD |
| *Lachnus roboris* | ACEA657-14 | BOLD |
| *Aphis mamonthovae* | ACEA658-14 | BOLD |
| *Hyperomyzus picridis* | ACEA659-14 | BOLD |
| *Aphis parietariae* | ACEA660-14 | BOLD |
| *Aphis fabae* | ACEA662-14 | BOLD |
| *Cavariella theobaldi* | ACEA664-14 | BOLD |
| *Dysaphis tulipae* | ACEA665-14 | BOLD |
| *Aphis sambuci* | ACEA666-14 | BOLD |
| *Dysaphis radicola* | ACEA667-14 | BOLD |
| *Aulacorthum solani* | ACEA668-14 | BOLD |
| *Brachycaudus cardui* | ACEA669-14 | BOLD |
| *Acyrthosiphon malvae* | ACEA671-14 | BOLD |
| *Acyrthosiphon pisum* | ACEA672-14 | BOLD |
| *Acyrthosiphon malvae* | ACEA673-14 | BOLD |
| *Aulacorthum solani* | ACEA674-14 | BOLD |
| *Aphis fabae* | ACEA675-14 | BOLD |
| *Aphis salicariae* | ACEA676-14 | BOLD |
| *Dysaphis apiifolia* | ACEA677-14 | BOLD |
| *Aphis galiiscabri* | ACEA678-14 | BOLD |
| *Dysaphis crataegi* | ACEA679-14 | BOLD |
| *Aphis craccivora* | ACEA680-14 | BOLD |
| *Sipha maydis* | ACEA681-14 | BOLD |
| *Aphis confusa* | ACEA682-14 | BOLD |
| *Nearctaphis bakeri* | ACEA683-14 | BOLD |
| *Uroleucon aeneum* | ACEA684-14 | BOLD |
| *Eucallipterus tiliae* | ACEA685-14 | BOLD |
| *Aphis proffti* | ACEA686-14 | BOLD |
| *Aphis fabae* | ACEA687-14 | BOLD |
| *Aphis epilobii* | ACEA688-14 | BOLD |
| *Aphis crepidis* | ACEA689-14 | BOLD |
| *Aphis pomi* | ACEA690-14 | BOLD |
| *Chaitophorus capreae* | ACEA691-14 | BOLD |
| *Aphis plantaginis* | ACEA692-14 | BOLD |
| *Aphis fabae* | ACEA693-14 | BOLD |
| *Aphis epilobii* | ACEA694-14 | BOLD |
| *Aphis oenotherae* | ACEA695-14 | BOLD |
| *Aphis pomi* | ACEA696-14 | BOLD |
| *Hayhurstia atriplicis* | ACEA697-14 | BOLD |
| *Aphis fabae* | ACEA698-14 | BOLD |
| *Aphis fabae* | ACEA699-14 | BOLD |
| *Uroleucon sonchi* | ACEA700-14 | BOLD |
| *Hyperomyzus lactucae* | ACEA701-14 | BOLD |
| *Aphis hederae* | ACEA702-14 | BOLD |
| *Dysaphis tulipae* | ACEA703-14 | BOLD |
| *Aphis grossulariae* | ACEA704-14 | BOLD |
| *Macrosiphum euphorbiae* | ACEA705-14 | BOLD |
| *Macrosiphoniella millefolii* | ACEA706-14 | BOLD |
| *Hyalopterus pruni* | ACEA707-14 | BOLD |
| *Aphis fabae* | ACEA708-14 | BOLD |
| *Hyperomyzus lactucae* | ACEA709-14 | BOLD |
| *Aphis plantaginis* | ACEA710-14 | BOLD |
| *Aphis mamonthovae* | ACEA711-14 | BOLD |
| *Aphis spiraecola* | ACEA712-14 | BOLD |
| *Chaitophorus leucomelas* | ACEA713-14 | BOLD |
| *Aphis pomi* | ACEA714-14 | BOLD |
| *Panaphis juglandis* | ACEA715-14 | BOLD |
| *Aphis gossypii* | ACEA716-14 | BOLD |
| *Semiaphis dauci* | ACEA717-14 | BOLD |
| *Aphis coronillae* | ACEA718-14 | BOLD |
| *Aphis coronillae* | ACEA719-14 | BOLD |
| *Aphis lambersi* | ACEA720-14 | BOLD |
| *Semiaphis dauci* | ACEA721-14 | BOLD |
| *Hydaphias molluginis* | ACEA722-14 | BOLD |
| *Roepkea marchali* | ACEA723-14 | BOLD |
| *Brachycaudus helichrysi* | ACEA724-14 | BOLD |
| *Myzus persicae* | ACEA725-14 | BOLD |
| *Myzus cerasi* | ACEA726-14 | BOLD |
| *Brachycaudus persicae* | ACEA727-14 | BOLD |
| *Myzus cerasi* | ACEA728-14 | BOLD |
| *Brachycaudus lychnidis* | ACEA729-14 | BOLD |
| *Brachycaudus amygdalinus* | ACEA730-14 | BOLD |
| *Hyalopterus amygdali* | ACEA731-14 | BOLD |
| *Brachycaudus amygdalinus* | ACEA732-14 | BOLD |
| *Myzus lythri* | ACEA733-14 | BOLD |
| *Brachycaudus tragopogonis* | ACEA734-14 | BOLD |
| *Brachycaudus helichrysi* | ACEA735-14 | BOLD |
| *Macrosiphum rosae* | ACEA736-14 | BOLD |
| *Aphis urticata* | ACEA737-14 | BOLD |
| *Brachycaudus helichrysi* | ACEA738-14 | BOLD |
| *Myzus cerasi* | ACEA739-14 | BOLD |
| *Brachycaudus schwartzi* | ACEA740-14 | BOLD |
| *Myzus cerasi* | ACEA741-14 | BOLD |
| *Aphis acetosae* | ACEA742-14 | BOLD |
| *Pterocomma populeum* | ACEA743-14 | BOLD |
| *Brachycaudus persicae* | ACEA744-14 | BOLD |
| *Brachycaudus schwartzi* | ACEA745-14 | BOLD |
| *Sitobion fragariae* | ACEA746-14 | BOLD |
| *Cryptomyzus ribis* | ACEA747-14 | BOLD |
| *Brachycaudus lateralis* | ACEA748-14 | BOLD |
| *Aulacorthum solani* | ACEA749-14 | BOLD |
| *Periphyllus testudinaceus* | ACEA750-14 | BOLD |
| *Brachycaudus populi* | ACEA751-14 | BOLD |
| *Brachycaudus tragopogonis* | ACEA752-14 | BOLD |
| *Brachycaudus lychnidis* | ACEA753-14 | BOLD |
| *Macrosiphum sileneum* | ACEA754-14 | BOLD |
| *Aphis fabae* | ACEA755-14 | BOLD |
| *Brachycaudus linariae* | ACEA756-14 | BOLD |
| *Brachycaudus cerinthis* | ACEA757-14 | BOLD |
| *Macrosiphum albifrons* | ACEA758-14 | BOLD |
| *Aphis idaei* | ACEA759-14 | BOLD |
| *Aphis pomi* | ACEA760-14 | BOLD |
| *Euceraphis betulae* | ACEA761-14 | BOLD |
| *Aphis grossulariae* | ACEA762-14 | BOLD |
| *Aphis epilobii* | ACEA763-14 | BOLD |
| *Aphis plantaginis* | ACEA764-14 | BOLD |
| *Aphis spiraephaga* | ACEA765-14 | BOLD |
| *Cinara fresai* | ACEA766-14 | BOLD |
| *Uroleucon jaceae* | ACEA767-14 | BOLD |
| *Hyadaphis passerinii* | ACEA768-14 | BOLD |
| *Brachycaudus aconiti* | ACEA769-14 | BOLD |
| *Dysaphis aucupariae* | ACEA770-14 | BOLD |
| *Dysaphis plantaginea* | ACEA771-14 | BOLD |
| *Brachycaudus lateralis* | ACEA772-14 | BOLD |
| *Brachycaudus helichrysi* | ACEA773-14 | BOLD |
| *Phorodon humuli* | ACEA774-14 | BOLD |
| *Rhopalosiphum nymphaeae* | ACEA775-14 | BOLD |
| *Brachycaudus helichrysi* | ACEA776-14 | BOLD |
| *Macrosiphum euphorbiae* | ACEA777-14 | BOLD |
| *Dysaphis plantaginea* | ACEA778-14 | BOLD |
| *Sipha elegans* | ACEA779-14 | BOLD |
| *Aulacorthum solani* | ACEA780-14 | BOLD |
| *Aphis intybi* | ACEA781-14 | BOLD |
| *Sipha maydis* | ACEA782-14 | BOLD |
| *Brachycaudus helichrysi* | ACEA783-14 | BOLD |
| *Brachycaudus linariae* | ACEA784-14 | BOLD |
| *Brachycaudus helichrysi* | ACEA785-14 | BOLD |
| *Neotoxoptera formosana* | ACEA786-14 | BOLD |
| *Aphis nerii* | ACEA787-14 | BOLD |
| *Macrosiphum rosae* | ACEA788-14 | BOLD |
| *Aphis nasturtii* | ACEA789-14 | BOLD |
| *Aploneura lentisci* | ACEA790-14 | BOLD |
| *Brachycaudus helichrysi* | ACEA791-14 | BOLD |
| *Aphis fabae* | ACEA792-14 | BOLD |
| *Myzus persicae* | ACEA793-14 | BOLD |
| *Rhopalosiphum maidis* | ACEA794-14 | BOLD |
| *Semiaphis dauci* | ACEA795-14 | BOLD |
| *Dysaphis foeniculus* | ACEA796-14 | BOLD |
| *Uroleucon inulae* | ACEA797-14 | BOLD |
| *Hyperomyzus lactucae* | ACEA798-14 | BOLD |
| *Brevicoryne brassicae* | ACEA799-14 | BOLD |
| *Therioaphis riehmi* | ACEA800-14 | BOLD |
| *Myzus persicae* | ACEA801-14 | BOLD |
| *Hoplocallis picta* | ACEA802-14 | BOLD |
| *Aphis cytisorum* | ACEA803-14 | BOLD |
| *Greenidea ficicola* | ACEA804-14 | BOLD |
| *Aphis fabae* | ACEA805-14 | BOLD |
| *Uroleucon sonchi* | ACEA806-14 | BOLD |
| *Lipaphis erysimi* | ACEA807-14 | BOLD |
| *Uroleucon sonchi* | ACEA808-14 | BOLD |
| *Eucallipterus tiliae* | ACEA809-14 | BOLD |
| *Myzocallis boerneri* | ACEA810-14 | BOLD |
| *Patchiella reaumuri* | ACEA811-14 | BOLD |
| *Cinara tujafilina* | ACEA812-14 | BOLD |
| *Cinara cedri* | ACEA813-14 | BOLD |
| *Cinara cedri* | ACEA814-14 | BOLD |
| *Cinara cedri* | ACEA815-14 | BOLD |
| *Myzus varians* | ACEA816-14 | BOLD |
| *Brachycaudus populi* | ACEA817-14 | BOLD |
| *Megoura viciae* | ACEA818-14 | BOLD |
| *Macrosiphum sileneum* | ACEA819-14 | BOLD |
| *Brachycolus cucubali* | ACEA820-14 | BOLD |
| *Brachycaudus tragopogonis* | ACEA821-14 | BOLD |
| *Aphis craccae* | ACEA822-14 | BOLD |
| *Brachycaudus rumexicolens* | ACEA823-14 | BOLD |
| *Brevicoryne brassicae* | ACEA824-14 | BOLD |
| *Brachycaudus amygdalinus* | ACEA825-14 | BOLD |
| *Brachycaudus cardui* | ACEA826-14 | BOLD |
| *Brachycaudus tragopogonis* | ACEA827-14 | BOLD |
| *Brachycaudus helichrysi* | ACEA828-14 | BOLD |
| *Myzocallis castanicola* | ACEA829-14 | BOLD |
| *Brachycaudus persicae* | ACEA830-14 | BOLD |
| *Brachycaudus prunicola* | ACEA831-14 | BOLD |
| *Thelaxes suberi* | ACEA832-14 | BOLD |
| *Aphis spiraecola* | ACEA833-14 | BOLD |
| *Aphis cytisorum* | ACEA834-14 | BOLD |
| *Brachycaudus cardui* | ACEA835-14 | BOLD |
| *Acyrthosiphon malvae* | ACEA836-14 | BOLD |
| *Aphis craccivora* | ACEA837-14 | BOLD |
| *Uroleucon aeneum* | ACEA838-14 | BOLD |
| *Anuraphis pyrilaseri* | ACEA839-14 | BOLD |
| *Panaphis juglandis* | ACEA840-14 | BOLD |
| *Anuraphis pyrilaseri* | ACEA841-14 | BOLD |
| *Euceraphis betulae* | ACEA842-14 | BOLD |
| *Euceraphis betulae* | ACEA843-14 | BOLD |
| *Drepanosiphum oregonensis* | ACEA844-14 | BOLD |
| *Brachycaudus populi* | ACEA845-14 | BOLD |
| *Aphis spiraecola* | ACEA846-14 | BOLD |
| *Macrosiphum sileneum* | ACEA847-14 | BOLD |
| *Eucarazzia elegans* | ACEA848-14 | BOLD |
| *Uroleucon hypochoeridis* | ACEA849-14 | BOLD |
| *Macrosiphum funestum* | ACEA850-14 | BOLD |
| *Aphis galiiscabri* | ACEA851-14 | BOLD |
| *Aphis fabae* | ACEA852-14 | BOLD |
| *Myzocallis coryli* | ACEA853-14 | BOLD |
| *Corylobium avellanae* | ACEA854-14 | BOLD |
| *Cinara pinimaritimae* | ACEA855-14 | BOLD |
| *Aphis lambersi* | ACEA856-14 | BOLD |
| *Macrosiphum cerinthiacum* | ACEA857-14 | BOLD |
| *Macrosiphoniella sanborni* | ACEA858-14 | BOLD |
| *Melanaphis donacis* | ACEA859-14 | BOLD |
| *Anuraphis pyrilaseri* | ACEA860-14 | BOLD |
| *Anuraphis pyrilaseri* | ACEA861-14 | BOLD |
| *Aphis verbasci* | ACEA862-14 | BOLD |
| *Appendiseta robiniae* | ACEA863-14 | BOLD |
| *Dysaphis tulipae* | ACEA864-14 | BOLD |
| *Aphis gossypii* | ACEA865-14 | BOLD |
| *Cavariella aegopodii* | ACEA866-14 | BOLD |
| *Lachnus roboris* | ACEA867-14 | BOLD |
| *Tuberculatus eggleri* | ACEA868-14 | BOLD |
| *Chaitophorus leucomelas* | ACEA869-14 | BOLD |
| *Aphis nerii* | ACEA870-14 | BOLD |
| *Aphis craccivora* | ACEA871-14 | BOLD |
| *Aphis spiraecola* | ACEA872-14 | BOLD |
| *Takecallis taiwana* | ACEA873-14 | BOLD |
| *Anthemidaphis ligusticae* | ACEA874-14 | BOLD |
| *Aphis fabae* | ACEA875-14 | BOLD |
| *Macrosiphoniella tanacetaria* | ACEA876-14 | BOLD |
| *Brachycaudus linariae* | ACEA877-14 | BOLD |
| *Brachycaudus helichrysi* | ACEA878-14 | BOLD |
| *Macrosiphum funestum* | ACEA879-14 | BOLD |
| *Anuraphis pyrilaseri* | ACEA880-14 | BOLD |
| *Uroleucon hypochoeridis* | ACEA881-14 | BOLD |
| *Anuraphis shaposhnikovi* | ACEA882-14 | BOLD |
| *Anuraphis subterranea* | ACEA883-14 | BOLD |
| *Melanaphis pyraria* | ACEA884-14 | BOLD |
| *Melanaphis pyraria* | ACEA885-14 | BOLD |
| *Macrosiphoniella millefolii* | ACEA886-14 | BOLD |
| *Prociphilus bumeliae* | ACEA887-14 | BOLD |
| *Periphyllus testudinaceus* | ACEA888-14 | BOLD |
| *Megoura viciae* | ACEA889-14 | BOLD |
| *Anuraphis subterranea* | ACEA890-14 | BOLD |
| *Crypturaphis grassii* | ACEA891-14 | BOLD |
| *Aphis punicae* | ACEA892-14 | BOLD |
| *Brachycaudus klugkisti* | ACEA893-14 | BOLD |
| *Brachycaudus klugkisti* | ACEA894-14 | BOLD |
| *Appendiseta robiniae* | ACEA895-14 | BOLD |
| *Tuberculatus eggleri* | ACEA896-14 | BOLD |
| *Aphis farinosa* | ACEA897-14 | BOLD |
| *Brachycaudus linariae* | ACEA898-14 | BOLD |
| *Brachycaudus klugkisti* | ACEA900-14 | BOLD |
| *Macrosiphum sileneum* | ACEA901-14 | BOLD |
| *Myzus persicae* | ACEA902-14 | BOLD |
| *Betulaphis quadrituberculata* | ACEA903-14 | BOLD |
| *Cavariella theobaldi* | ACEA904-14 | BOLD |
| *Megoura viciae* | ACEA905-14 | BOLD |
| *Macrosiphum rosae* | ACEA906-14 | BOLD |
| *Hyadaphis passerinii* | ACEA907-14 | BOLD |
| *Cavariella pastinacae* | ACEA908-14 | BOLD |
| *Hyperomyzus lactucae* | ACEA909-14 | BOLD |
| *Cavariella theobaldi* | ACEA910-14 | BOLD |
| *Hyalopterus pruni* | ACEA911-14 | BOLD |
| *Macrosiphum rosae* | ACEA912-14 | BOLD |
| *Macrosiphum euphorbiae* | ACEA913-14 | BOLD |
| *Brachycaudus helichrysi* | ACEA914-14 | BOLD |
| *Brachycaudus spiraeae* | ACEA915-14 | BOLD |
| *Uroleucon jaceae* | ACEA916-14 | BOLD |
| *Aphis fabae* | ACEA917-14 | BOLD |
| *Amphorophora rubi* | ACEA918-14 | BOLD |
| *Euceraphis punctipennis* | ACEA919-14 | BOLD |
| *Tuberculatus annulatus* | ACEA920-14 | BOLD |
| *Aphis taraxacicola* | ACEA921-14 | BOLD |
| *Aphis craccae* | ACEA922-14 | BOLD |
| *Aphis salicariae* | ACEA923-14 | BOLD |
| *Aphis fabae* | ACEA924-14 | BOLD |
| *Brachycaudus sedi* | ACEA925-14 | BOLD |
| *Tuberculatus annulatus* | ACEA926-14 | BOLD |
| *Aphis cytisorum* | ACEA927-14 | BOLD |
| *Pemphigus vesicarius* | ACEA928-14 | BOLD |
| *Chaitophorus leucomelas* | ACEA929-14 | BOLD |
| *Aphis intybi* | ACEA930-14 | BOLD |
| *Aphis confusa* | ACEA931-14 | BOLD |
| *Brachycaudus tragopogonis* | ACEA932-14 | BOLD |
| *Dysaphis apiifolia* | ACEA933-14 | BOLD |
| *Macrosiphum rosae* | ACEA934-14 | BOLD |
| *Aphis nerii* | ACEA935-14 | BOLD |
| *Aphis ruborum* | ACEA936-14 | BOLD |
| *Eriosoma lanuginosum* | ACEA937-14 | BOLD |
| *Brachycaudus cardui* | ACEA938-14 | BOLD |
| *Metopeurum fuscoviride* | ACEA939-14 | BOLD |
| *Aphis spiraecola* | ACEA940-14 | BOLD |
| *Brachycaudus helichrysi* | ACEA941-14 | BOLD |
| *Brachycaudus helichrysi* | ACEA942-14 | BOLD |
| *Brachycaudus helichrysi* | ACEA943-14 | BOLD |
| *Brachycaudus helichrysi* | ACEA944-14 | BOLD |
| *Brachycaudus helichrysi* | ACEA945-14 | BOLD |
| *Brachycaudus lychnidis* | ACEA946-14 | BOLD |
| *Uroleucon aeneum* | ACEA947-14 | BOLD |
| *Metopolophium dirhodum* | ACEA948-14 | BOLD |
| *Sipha maydis* | ACEA949-14 | BOLD |
| *Aphis confusa* | ACEA950-14 | BOLD |
| *Hyperomyzus lactucae* | ACEA951-14 | BOLD |
| *Eriosoma lanigerum* | ACEA952-14 | BOLD |
| *Brachycaudus helichrysi* | ACEA953-14 | BOLD |
| *Brachycaudus helichrysi* | ACEA954-14 | BOLD |
| *Brachycaudus helichrysi* | ACEA955-14 | BOLD |
| *Brachycaudus amygdalinus* | ACEA956-14 | BOLD |
| *Rhopalosiphum padi* | ACEA957-14 | BOLD |
| *Myzus persicae* | ACEA958-14 | BOLD |
| *Takecallis arundinariae* | ACEA959-14 | BOLD |
| *Melanaphis bambusae* | ACEA960-14 | BOLD |
| *Brachycaudus helichrysi* | ACEA961-14 | BOLD |
| *Wahlgreniella arbuti* | ACEA962-14 | BOLD |
| *Sipha maydis* | ACEA963-14 | BOLD |
| *Brachycaudus helichrysi* | ACEA964-14 | BOLD |
| *Macrosiphum euphorbiae* | ACEA965-14 | BOLD |
| *Takecallis arundicolens* | ACEA966-14 | BOLD |
| *Uroleucon inulae* | ACEA967-14 | BOLD |
| *Megoura viciae* | ACEA968-14 | BOLD |
| *Hyalopterus amygdali* | ACEA969-14 | BOLD |
| *Aphis vallei* | ACEA970-14 | BOLD |
| *Myzus persicae* | ACEA971-14 | BOLD |
| *Tinocallis takachihoensis* | ACEA972-14 | BOLD |
| *Aphis viticis* | ACEA973-14 | BOLD |
| *Brachycaudus helichrysi* | ACEA974-14 | BOLD |
| *Macrosiphum euphorbiae* | ACEA975-14 | BOLD |
| *Brevicoryne brassicae* | ACEA976-14 | BOLD |
| *Sipha maydis* | ACEA977-14 | BOLD |
| *Aphis lugentis* | ACEA978-14 | BOLD |
| *Aphis umbrella* | ACEA979-14 | BOLD |
| *Brachycaudus helichrysi* | ACEA980-14 | BOLD |
| *Hyadaphis foeniculi* | ACEA981-14 | BOLD |
| *Brachycaudus amygdalinus* | ACEA982-14 | BOLD |
| *Brachycaudus helichrysi* | ACEA983-14 | BOLD |
| *Melanaphis donacis* | ACEA984-14 | BOLD |
| *Ceruraphis eriophori* | ACEA985-14 | BOLD |
| *Brachycaudus helichrysi* | ACEA986-14 | BOLD |
| *Brachycaudus helichrysi* | ACEA987-14 | BOLD |
| *Brachycaudus helichrysi* | ACEA988-14 | BOLD |
| *Brachycaudus helichrysi* | ACEA989-14 | BOLD |
| *Cinara confinis* | ACEA990-14 | BOLD |
| *Elatobium abietinum* | ACEA991-14 | BOLD |
| *Brachycaudus helichrysi* | ACEA992-14 | BOLD |
| *Brachycaudus helichrysi* | ACEA993-14 | BOLD |
| *Brachycaudus helichrysi* | ACEA994-14 | BOLD |
| *Brachycaudus helichrysi* | ACEA995-14 | BOLD |
| *Dysaphis plantaginea* | ACEA996-14 | BOLD |
| *Myzus cerasi* | ACEA997-14 | BOLD |
| *Brachycaudus helichrysi* | ACEA998-14 | BOLD |
| *Brachycaudus helichrysi* | ACEA999-14 | BOLD |
| *Aphis schneideri* | DQ418823.1 | GenBank |
| *Aphis schneideri* | DQ418824.1 | GenBank |
| *Aphis schneideri* | DQ418825.1 | GenBank |
| *Aphis oenotherae* | DQ418837.1 | GenBank |
| *Aphis oenotherae* | DQ418838.1 | GenBank |
| *Aphis oenotherae* | DQ418839.1 | GenBank |
| *Macrosiphum rosae* | DQ499035.1 | GenBank |
| *Myzus ornatus* | DQ499044.1 | GenBank |
| *Brachycaudus populi* | EU189642.1 | GenBank |
| *Brachycaudus lychnidis* | EU189649.1 | GenBank |
| *Brachycaudus lychnidis* | EU189664.1 | GenBank |
| *Brachycaudus napelli* | EU189695.1 | GenBank |
| *Brachycaudus napelli* | EU196589.1 | GenBank |
| *Brachycaudus napelli* | EU196613.1 | GenBank |
| *Brachycorynella asparagus* | EU701536.1 | GenBank |
| *Liosomaphis berberi* | EU701709.1 | GenBank |
| *Metopeurum fuscoviride* | EU701751.1 | GenBank |
| *Myzus ascalonicus* | EU701782.1 | GenBank |
| *Myzus ornatus* | EU701794.1 | GenBank |
| *Periphyllus lyropic* | EU701850.1 | GenBank |
| *Rhopalosiphum insertum* | EU701888.1 | GenBank |
| *Rhopalosiphum insertum* | EU701889.1 | GenBank |
| *Cryptosiphum artemi* | FJ965599.1 | GenBank |
| *Cryptosiphum artemi* | GU457800.1 | GenBank |
| *Longicaudus trirhod* | GU978804.1 | GenBank |
| *Brachycaudus aconiti* | JF340110.1 | GenBank |
| *Sitobion fragariae* | JX507420.1 | GenBank |
| *Microlophium carnosum* | JX507422.1 | GenBank |
| *Hyalopteroides humilis* | JX507428.1 | GenBank |
| *Longicaudus trirhod* | JX844377.1 | GenBank |
| *Longicaudus trirhod* | JX844385.1 | GenBank |
| *Wahlgreniella nerva* | KF285590.1 | GenBank |
| *Acyrthosiphon malvae* | KF638722.1 | GenBank |
| *Acyrthosiphon malvae* | KF638723.1 | GenBank |
| *Acyrthosiphon malvae* | KF638724.1 | GenBank |
| *Acyrthosiphon pisum* | KF638725.1 | GenBank |
| *Acyrthosiphon pisum* | KF638726.1 | GenBank |
| *Amphorophora rubi* | KF638730.1 | GenBank |
| *Amphorophora rubi* | KF638731.1 | GenBank |
| *Amphorophora rubi* | KF638732.1 | GenBank |
| *Anoecia corni* | KF638733.1 | GenBank |
| *Aphis chloris* | KF638752.1 | GenBank |
| *Aphis nasturtii* | KF638968.1 | GenBank |
| *Aphis nasturtii* | KF638969.1 | GenBank |
| *Aphis ruborum* | KF639019.1 | GenBank |
| *Aphis ruborum* | KF639021.1 | GenBank |
| *Aphis rumicis* | KF639022.1 | GenBank |
| *Aphis rumicis* | KF639023.1 | GenBank |
| *Aphis sambuci* | KF639030.1 | GenBank |
| *Aphis sambuci* | KF639033.1 | GenBank |
| *Aphis sambuci* | KF639034.1 | GenBank |
| *Aphis spiraecola* | KF639075.1 | GenBank |
| *Aphis spiraecola* | KF639076.1 | GenBank |
| *Aphis ulmariae* | KF639087.1 | GenBank |
| *Aphis ulmariae* | KF639088.1 | GenBank |
| *Aphis umbrella* | KF639089.1 | GenBank |
| *Aphis umbrella* | KF639090.1 | GenBank |
| *Aphis umbrella* | KF639091.1 | GenBank |
| *Aphis urticata* | KF639095.1 | GenBank |
| *Aphis urticata* | KF639096.1 | GenBank |
| *Aphis urticata* | KF639097.1 | GenBank |
| *Aphis verbasci* | KF639105.1 | GenBank |
| *Aphis verbasci* | KF639106.1 | GenBank |
| *Aphis verbasci* | KF639107.1 | GenBank |
| *Aulacorthum solani* | KF639121.1 | GenBank |
| *Aulacorthum solani* | KF639122.1 | GenBank |
| *Aulacorthum solani* | KF639123.1 | GenBank |
| *Brachycaudus aconiti* | KF639127.1 | GenBank |
| *Brachycaudus cardui* | KF639150.1 | GenBank |
| *Brachycaudus cardui* | KF639151.1 | GenBank |
| *Brachycaudus cardui* | KF639152.1 | GenBank |
| *Brachycaudus helichrysi* | KF639193.1 | GenBank |
| *Brachycaudus helichrysi* | KF639194.1 | GenBank |
| *Brachycaudus lateralis* | KF639204.1 | GenBank |
| *Brachycaudus lateralis* | KF639205.1 | GenBank |
| *Brachycaudus lateralis* | KF639206.1 | GenBank |
| *Brevicoryne brassicae* | KF639254.1 | GenBank |
| *Brevicoryne brassicae* | KF639255.1 | GenBank |
| *Brevicoryne brassicae* | KF639256.1 | GenBank |
| *Ceruraphis eriophori* | KF639273.1 | GenBank |
| *Ceruraphis eriophori* | KF639274.1 | GenBank |
| *Ceruraphis eriophori* | KF639275.1 | GenBank |
| *Hyadaphis foeniculi* | KF639403.1 | GenBank |
| *Hyadaphis foeniculi* | KF639404.1 | GenBank |
| *Hyadaphis foeniculi* | KF639405.1 | GenBank |
| *Hyalopterus pruni* | KF639420.1 | GenBank |
| *Hyalopterus pruni* | KF639421.1 | GenBank |
| *Hyalopterus pruni* | KF639422.1 | GenBank |
| *Hyperomyzus lactucae* | KF639438.1 | GenBank |
| *Hyperomyzus lactucae* | KF639439.1 | GenBank |
| *Hyperomyzus lactucae* | KF639440.1 | GenBank |
| *Lipaphis erysimi* | KF639452.1 | GenBank |
| *Lipaphis erysimi* | KF639453.1 | GenBank |
| *Macrosiphoniella absinthii* | KF639457.1 | GenBank |
| *Macrosiphoniella artemisiae* | KF639458.1 | GenBank |
| *Macrosiphoniella artemisiae* | KF639459.1 | GenBank |
| *Macrosiphoniella millefolii* | KF639461.1 | GenBank |
| *Macrosiphoniella millefolii* | KF639462.1 | GenBank |
| *Macrosiphoniella millefolii* | KF639463.1 | GenBank |
| *Macrosiphoniella oblonga* | KF639464.1 | GenBank |
| *Macrosiphum euphorbiae* | KF639489.1 | GenBank |
| *Megoura viciae* | KF639516.1 | GenBank |
| *Metopeurum fuscoviride* | KF639531.1 | GenBank |
| *Metopeurum fuscoviride* | KF639532.1 | GenBank |
| *Microlophium carnosum* | KF639535.1 | GenBank |
| *Myzus cerasi* | KF639553.1 | GenBank |
| *Myzus cerasi* | KF639554.1 | GenBank |
| *Myzus cerasi* | KF639555.1 | GenBank |
| *Myzus persicae* | KF639566.1 | GenBank |
| *Myzus persicae* | KF639567.1 | GenBank |
| *Myzus persicae* | KF639568.1 | GenBank |
| *Rhopalosiphum insertum* | KF639623.1 | GenBank |
| *Rhopalosiphum nymphaeae* | KF639625.1 | GenBank |
| *Rhopalosiphum padi* | KF639626.1 | GenBank |
| *Rhopalosiphum padi* | KF639627.1 | GenBank |
| *Uroleucon achilleae* | KF639678.1 | GenBank |
| *Uroleucon aeneum* | KF639679.1 | GenBank |
| *Uroleucon aeneum* | KF639681.1 | GenBank |
| *Uroleucon aeneum* | KF639682.1 | GenBank |
| *Uroleucon hypochoeridis* | KF639696.1 | GenBank |
| *Uroleucon hypochoeridis* | KF639701.1 | GenBank |
| *Uroleucon jaceae* | KF639712.1 | GenBank |
| *Uroleucon jaceae* | KF639714.1 | GenBank |
| *Uroleucon jaceae* | KF639715.1 | GenBank |
| *Uroleucon nigrocampanulae* | KF639716.1 | GenBank |
| *Rhopalosiphum nymphaeae* | KJ722008.1 | GenBank |
| *Rhopalosiphum nymphaeae* | KJ722009.1 | GenBank |
| *Sitobion fragariae* | KM376049.1 | GenBank |
| *Periphyllus hirtico* | KR029859.1 | GenBank |
| *Macrosiphoniella artemisiae* | KR029909.1 | GenBank |
| *Macrosiphoniella oblonga* | KR029917.1 | GenBank |
| *Microlophium carnosum* | KR029948.1 | GenBank |
| *Sitobion fragariae* | KR029952.1 | GenBank |
| *Aphis oenotherae* | KR031111.1 | GenBank |
| *Myzus ascalonicus* | KR031197.1 | GenBank |
| *Rhopalosiphoninus staphyleae* | KR031605.1 | GenBank |
| *Liosomaphis berberi* | KR033543.1 | GenBank |
| *Macrosiphoniella absinthii* | KR033941.1 | GenBank |
| *Amphorophora ampullata* | KR034838.1 | GenBank |
| *Chaetosiphon fragaefolii* | KR035154.1 | GenBank |
| *Myzus ascalonicus* | KR035159.1 | GenBank |
| *Aphis oenotherae* | KR036309.1 | GenBank |
| *Macrosiphoniella tanacetaria* | KR039542.1 | GenBank |
| *Macrosiphoniella absinthii* | KR040544.1 | GenBank |
| *Liosomaphis berberi* | KR040620.1 | GenBank |
| *Amphorophora ampullata* | KR040891.1 | GenBank |
| *Aphis fabae* | KR042004.1 | GenBank |
| *Macrosiphoniella tanacetaria* | KR042085.1 | GenBank |
| *Myzus ornatus* | KR042195.1 | GenBank |
| *Aphis oenotherae* | KR043103.1 | GenBank |
| *Macrosiphum rosae* | KR043704.1 | GenBank |
| *Coloradoa absinthii* | KR044231.1 | GenBank |
| *Macrosiphum rosae* | KR044630.1 | GenBank |
| *Periphyllus testudinaceus* | KR341594.1 | GenBank |
| *Periphyllus testudinaceus* | KR342462.1 | GenBank |
| *Periphyllus testudinaceus* | KR562707.1 | GenBank |
| *Schizaphis graminum* | KR572162.1 | GenBank |
| *Amphorophora ampullata* | KR572844.1 | GenBank |
| *Chaetosiphon fragaefolii* | KR573016.1 | GenBank |
| *Chaetosiphon fragaefolii* | KR573418.1 | GenBank |
| *Amphorophora ampullata* | KR573598.1 | GenBank |
| *Amphorophora ampullata* | KR574604.1 | GenBank |
| *Macrosiphum rosae* | KR574868.1 | GenBank |
| *Microlophium carnosum* | KR575734.1 | GenBank |
| *Periphyllus testudinaceus* | KR581272.1 | GenBank |
| *Macrosiphoniella tanacetaria* | KR581870.1 | GenBank |
| *Periphyllus testudinaceus* | KR583502.1 | GenBank |
| *Hyalopteroides humilis* | KR583738.1 | GenBank |
| *Periphyllus testudinaceus* | KR583758.1 | GenBank |
| *Hyalopteroides humilis* | KR917803.1 | GenBank |
| *Wahlgreniella nerva* | KU567955.1 | GenBank |
| *Wahlgreniella nerva* | KU567956.1 | GenBank |
| *Periphyllus hirtico* | KU570426.1 | GenBank |
| *Sitobion avenae* | KX054740.1 | GenBank |
| *Pleotrichophorus glandulosus* | KX631494.1 | GenBank |
| *Idiopterus nephrele* | KX631545.1 | GenBank |
| *Chromaphis juglandicola* | KY306845.1 | GenBank |
| *Chromaphis juglandicola* | KY306846.1 | GenBank |
| *Chromaphis juglandicola* | KY306847.1 | GenBank |
| *Therioaphis trifolii* | KY307074.1 | GenBank |
| *Tuberculatus annulatus* | KY307301.1 | GenBank |
| *Tuberculatus annulatus* | KY307303.1 | GenBank |
| *Tuberculatus annulatus* | KY307304.1 | GenBank |
| *Periphyllus lyropic* | KY832398.1 | GenBank |
| *Acyrthosiphon pisum* | KY846818.1 | GenBank |
| *Sitobion avenae* | MF154384.1 | GenBank |
| *Sitobion avenae* | MF154385.1 | GenBank |
| *Sitobion avenae* | MF154386.1 | GenBank |
| *Megoura viciae* | MF458754.1 | GenBank |
| *Uroleucon achilleae* | MG163862.1 | GenBank |
| *Sitobion avenae* | MG164365.1 | GenBank |
| *Macrosiphum rosae* | MG167708.1 | GenBank |
| *Hyalopteroides humilis* | MG168014.1 | GenBank |
| *Aphis fabae* | MG168411.1 | GenBank |
| *Uroleucon achilleae* | MG169020.1 | GenBank |
| *Rhopalosiphum padi* | MG169684.1 | GenBank |
| *Schizaphis graminum* | MG169839.1 | GenBank |
| *Schizaphis graminum* | MG169956.1 | GenBank |
| *Schizaphis graminum* | MG170403.1 | GenBank |
| *Therioaphis trifolii* | MG170412.1 | GenBank |
| *Therioaphis trifolii* | MG170996.1 | GenBank |
