# Supplementary material for: Insights into aphid prey consumption by ladybirds: Optimising field sampling methods and primer design for high throughput sequencing
Source: PLoS One. 2020 Jul 1;15(7):e0235054. doi: 10.1371/journal.pone.0235054 (PMC7329105; doi:10.1371/journal.pone.0235054)
Supplement: S2 Table — (DOCX) [file pone.0235054.s002.docx]

| **Landscape** | **Code** | **Coordinate system** | **Easting** | **Northing** | **Zone** | **Country** |
| --- | --- | --- | --- | --- | --- | --- |
| Schafisheim (AG) 5503 | L1 | UTM | 435177 | 5248045 | 32T | Switzerland |
| Stetten (AG) 5608 | L2 | UTM | 446470 | 5250416 | 32T | Switzerland |
| Bellikon (AG) 5454 | L3 | UTM | 450011 | 5249789 | 32T | Switzerland |
| Untersiggenthal (AG) 5417 | L4 | UTM | 445167 | 5262170 | 32T | Switzerland |
| Steinmaur (ZH) 8162 | L5 | UTM | 459011 | 5261365 | 32T | Switzerland |
| Will (ZH) 8196 | L6 | UTM | 463653 | 5272890 | 32T | Switzerland |
| Volken (ZH) 8459 | L7 | UTM | 471721 | 5268743 | 32T | Switzerland |
| Embrach (ZH) 8424 | L8 | UTM | 470504 | 5260995 | 32T | Switzerland |
| Rikon (ZH) 8486 | L9 | UTM | 484171 | 5254499 | 32T | Switzerland |
| Felben-Wellhausen (TG) 8552 | L10 | UTM | 496700 | 5270278 | 32T | Switzerland |
| Berg (TG) 5872 | L11 | UTM | 512175 | 5268533 | 32T | Switzerland |
| Bürglen (TG) 8575 | L12 | UTM | 511018 | 5264910 | 32T | Switzerland |
| Offenbach1 | Offenbach1 | UTM | 440979 | 5449520 | 32N | Germany |
| Offenbach2 | Offenbach2 | UTM | 439862 | 5448812 | 32N | Germany |
| Gommersheim1 | Gommersheim 1 | UTM | 449059 | 5459725 | 32N | Germany |
| Gommersheim2 | Gommersheim 2 | UTM | 447411 | 5459365 | 32N | Germany |
| Weingarten | Weingarten | UTM | 448171 | 5457133 | 32N | Germany |
| Schwegenheim | Schwegenheim | UTM | 452743 | 5458773 | 32N | Germany |
| Kandel1 | Kandel 1 | UTM | 441893 | 5437808 | 32N | Germany |
| Kandel2 | Kandel 2 | UTM | 440837 | 5439736 | 32N | Germany |
| KandelZ | Kandel Z | UTM | 443297 | 5439006 | 32N | Germany |
| Waghäusel2 | Waghäusel 2 | UTM | 463980 | 5450887 | 32N | Germany |
| Waghäusel1 | Waghäusel 1 | UTM | 464702 | 5451518 | 32N | Germany |

**S2 Table. Central coordinates of landscape sectors**
