# Supplementary material for: Insights into aphid prey consumption by ladybirds: Optimising field sampling methods and primer design for high throughput sequencing
Source: PLoS One. 2020 Jul 1;15(7):e0235054. doi: 10.1371/journal.pone.0235054 (PMC7329105; doi:10.1371/journal.pone.0235054)
Supplement: S3 Table — (DOCX) [file pone.0235054.s003.docx]

**S3 Table.** **Sampling rounds and corresponding dates.**

| Round | Time interval | Dates |
| --- | --- | --- |
| R1 | Int 1 | 01/04/16 - 14/04/16 |
| R2 | Int 1 | 15/04/16 - 27/04/16 |
| R3 | Int 2 | 28/04/16 - 08/05/16 |
| R4 | Int 2 | 09/05/16 - 19/05/16 |
| R5 | Int 3 | 20/05/16 - 30/05/16 |
| R6 | Int 3 | 31/05/16 - 13/06/16 |
| R7 | Int 4 | 14/06/16 - 26/06/16 |
| R8 | Int 4 | 27/06/16 - 12/07/16 |
