# Supplementary material for: Insights into aphid prey consumption by ladybirds: Optimising field sampling methods and primer design for high throughput sequencing
Source: PLoS One. 2020 Jul 1;15(7):e0235054. doi: 10.1371/journal.pone.0235054 (PMC7329105; doi:10.1371/journal.pone.0235054)
Supplement: S4 Table — (DOCX) [file pone.0235054.s004.docx]

**S4 Table. Aphid OTU and species information retrieved from ladybird samples.**

| Aphid taxa | Aphid reads | OTU per taxon | Occurrence in  *C. septempuctata* | Occurrence in *H. axyridis* |
| --- | --- | --- | --- | --- |
| *Acyrthosiphon caraganae* | 17 | 1 | 1 | 0 |
| *Acyrthosiphon malvae* | 1968 | 2 | 9 | 1 |
| *Acyrthosiphon pisum* | 22 | 1 | 1 | 0 |
| *Aphis spp.* | 15057 | 10 | 24 | 18 |
| *Aulacorthum solani* | 17 | 1 | 1 | 0 |
| *Brachycaudus spp.* | 1471 | 2 | 2 | 0 |
| *Chaetosiphon fragaefolii* | 429 | 1 | 3 | 0 |
| *Chromaphis juglandicola* | 770 | 1 | 0 | 2 |
| *Drepanosiphum oregonensis* | 556 | 1 | 0 | 1 |
| *Hyperomyzus lactucae* | 59 | 1 | 0 | 1 |
| *Laingia psammae* | 258 | 1 | 1 | 0 |
| *Macrosiphoniella artemisiae* | 37 | 1 | 1 | 0 |
| *Macrosiphoniella tanacetaria* | 14 | 1 | 1 | 0 |
| *Macrosiphum spp.* | 1603 | 3 | 3 | 2 |
| *Microlophium carnosum* | 45492 | 2 | 31 | 64 |
| *Myzus cerasi* | 2023 | 2 | 6 | 0 |
| *Periphyllus testudinaceus* | 4548 | 3 | 1 | 4 |
| *Pterocomma pilosum* | 205 | 1 | 0 | 1 |
| *Rhopalosiphum padi* | 383 | 1 | 5 | 1 |
| *Sitobion avenae* | 4089 | 3 | 13 | 3 |
| *Tuberculatus annulatus* | 259 | 1 | 0 | 1 |
| *Uroleucon achilleae* | 123 | 1 | 0 | 1 |
| *Uroleucon hypochoeridis* | 4363 | 1 | 2 | 0 |
| *Wahlgreniella spp.* | 52 | 1 | 0 | 1 |
| **Total** | 83815 | 43 | 105 | 101 |
