# Supplementary figures and images for: Insights into aphid prey consumption by ladybirds: Optimising field sampling methods and primer design for high throughput sequencing
Source: PLoS One. 2020 Jul 1;15(7):e0235054. doi: 10.1371/journal.pone.0235054 (PMC7329105; doi:10.1371/journal.pone.0235054)

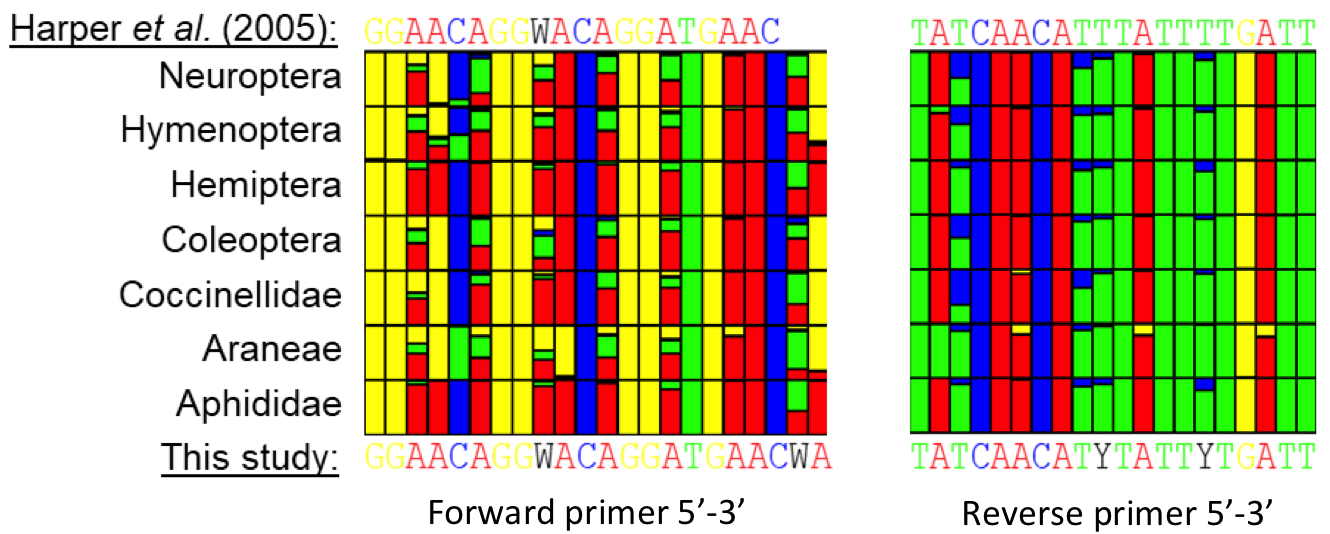

Supplement: S1 Fig — Primers modified for this study and those used by Harper et al. (2005) aligned with mass-alignments of taxa downloaded via PrimerMiner. The forward primer is on the left, the reverse primer on the right, both sequence alignments are oriented in 5’– 3’ direction. (TIF) [file pone.0235054.s005.tif]

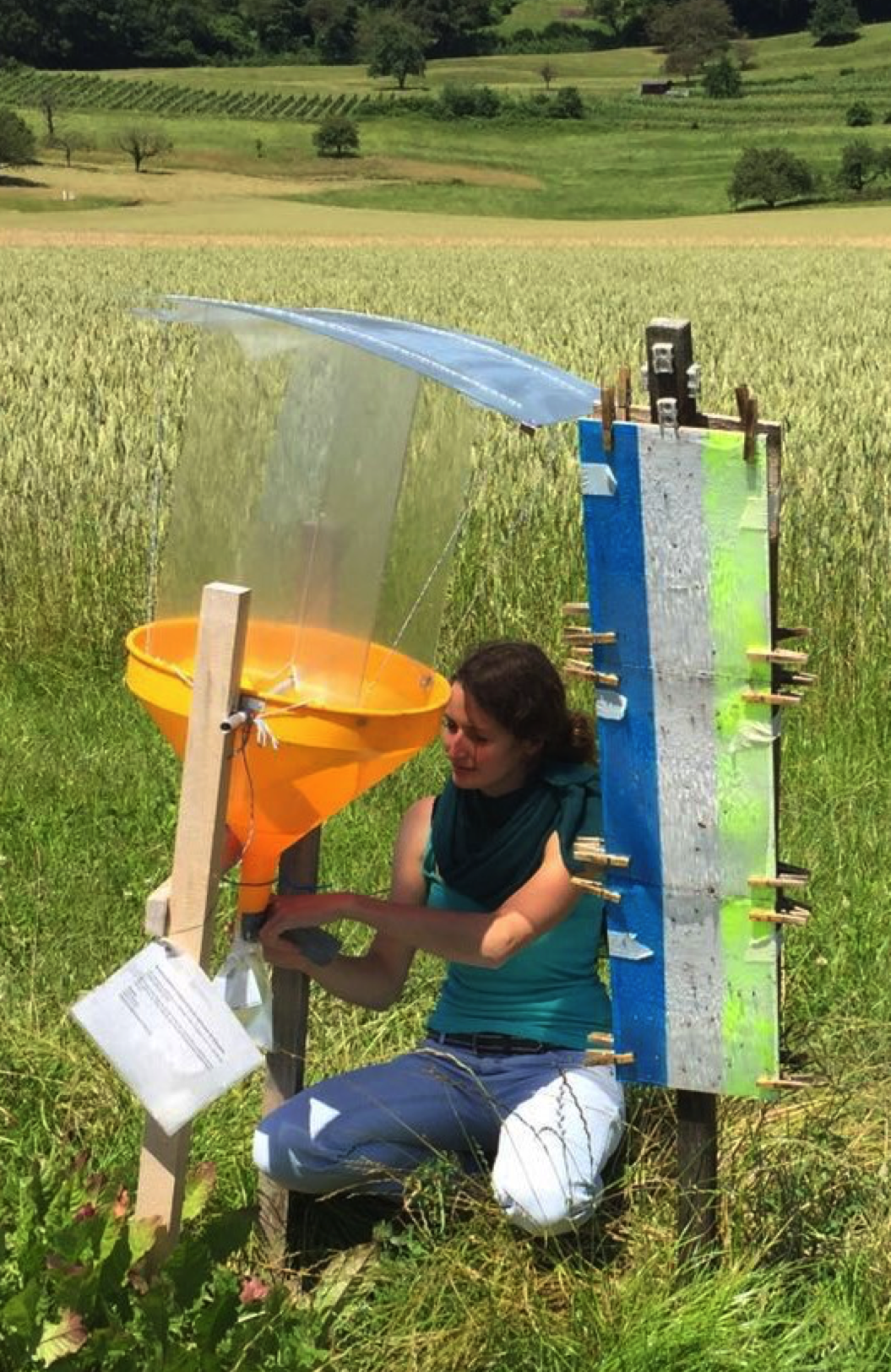

Supplement: S2 Fig — The combi trap (yellow, 42.5 cm upper diameter) has a whirl-pack® bag (Sigma-Aldrich) attached on the bottom, filled with 95% ethanol and a roof on top to prevent dilution through rain. For the sticky trap (891cm x 210cm), foils (Folex Foils Laserptinter BG-64 from OfficeWorld Switzerland) are attached with clothes pegs and/or tape to the coloured board (yellow, blue, white; Sparvar UV reflecting colour of Spray-Color GmbH) and were then sprayed with glue (Soveurode spray glue from Witasek, Austria). (TIF) [file pone.0235054.s006.tif]

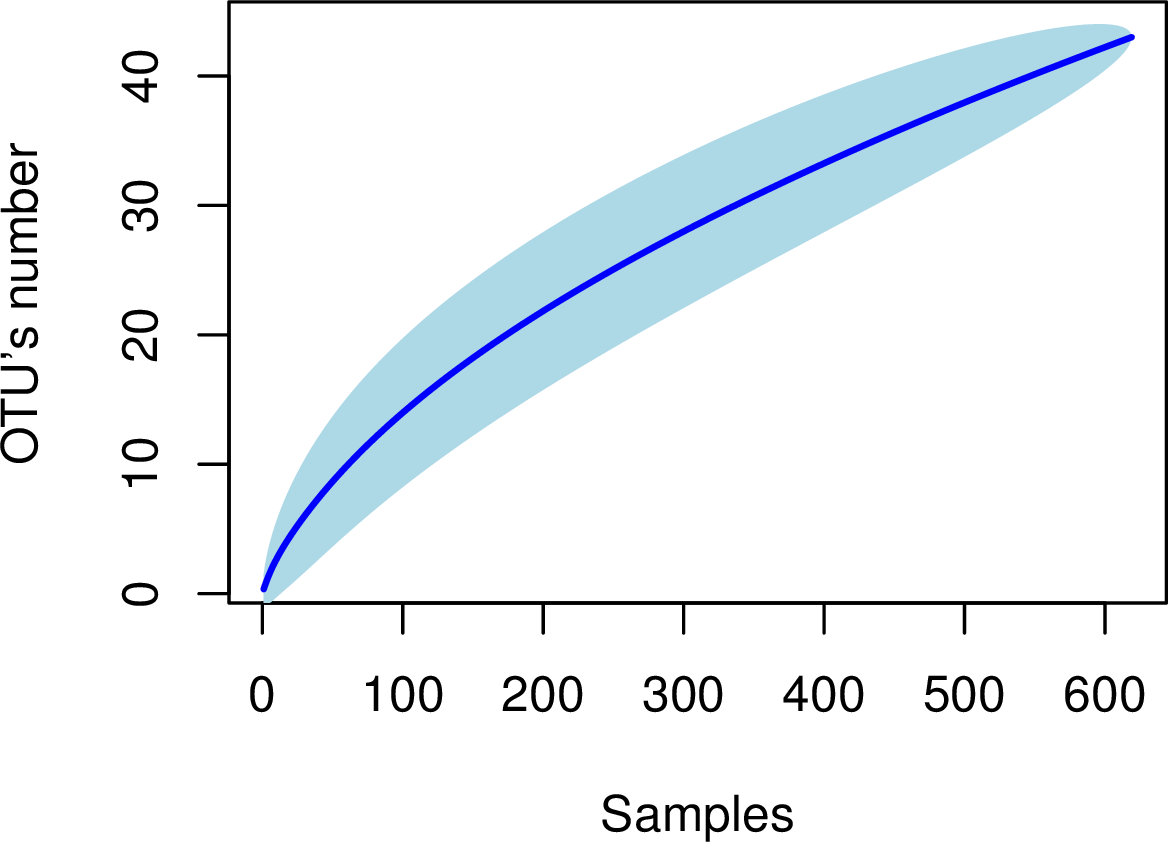

Supplement: S3 Fig — Curvature index: y = 0.5826x0.675 on 619 samples. (TIF) [file pone.0235054.s007.tif]

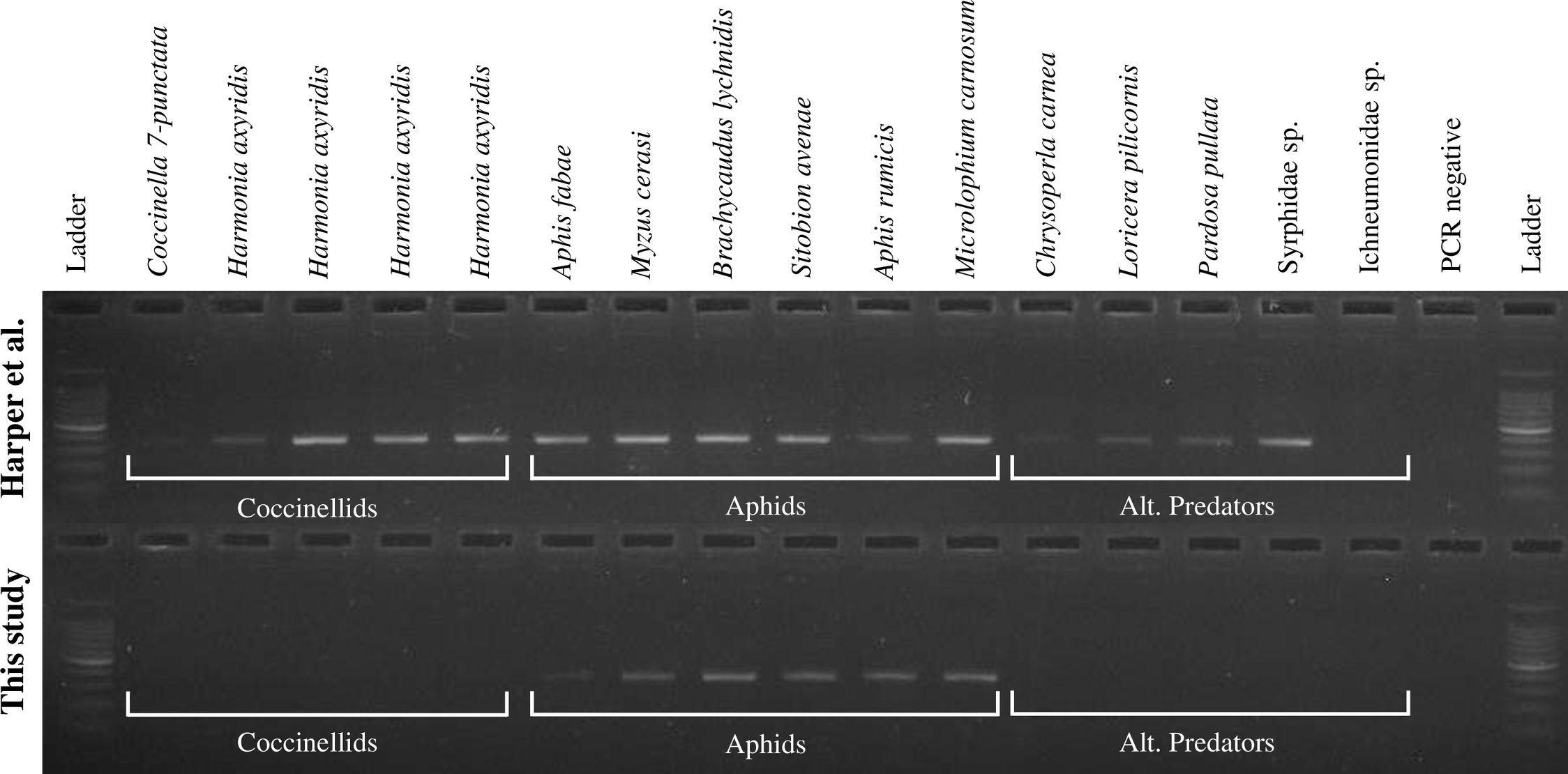

Supplement: S4 Fig — The modified primers of this study show increased specifity towards target aphid DNA, missing to amplify ladybird DNA. (TIF) [file pone.0235054.s008.tif]
