# Supplementary material for: Insights into aphid prey consumption by ladybirds: Optimising field sampling methods and primer design for high throughput sequencing
Source: PLoS One. 2020 Jul 1;15(7):e0235054. doi: 10.1371/journal.pone.0235054 (PMC7329105; doi:10.1371/journal.pone.0235054)
Supplement: S1 Appendix — (DOCX) [file pone.0235054.s011.docx]

**S1 Appendix. Details on bioinformatics procedures**

This supplementary document contains information for demultiplexing samples, including the necessary skripts and detailed procedures for dealing with tag-jumping.

**Demultiplexing**

This section includes two sets of perl scripts to demultiplex the data following the previous processing steps in Mothur v1.37.1 (41). Both perl scripts are edited for use with Pool 1. The four associated text files required to complete these processing steps on Pool 1 and Pool 2 are located in the supplementary material called “S5_P1SampleList1.txt” and “S5_P1SampleList2.txt” for Pool 1 and “S5_P2SampleList1.txt” and “S5_P2SampleList2.txt” for Pool2.

**Script 1 “deplexstep1.pl” :**

#!/usr/bin/perl

unless ($#ARGV == 0)

{

print "Usage: deplexstep1.pl S5_P1SampleList1.txt";

die;

}

open (INLIST, "<$ARGV[0]") || die;

# replace 'XXX' with your input and output directories

$indir = " XXX ";

$outdir = “XXX ";

# Loops through the list of your samples (in 'S5_P1SampleList1.txt ') and performs the commands for each one

while (<INLIST>) {

$lib = $_;

chomp($lib);

# A shortcut to read or write a file for each of your samples, each file having the same extension

$readids1 = $lib . "_ids.txt";

$fa1 = $lib . ".fasta";

# split fasta read IDs into files grouped by sample ID. Replace 'XX' with the name of the '.groups' file (output from mothur)

system("grep -w $lib $indir/XX.groups | awk '{print \$1}' > $outdir/$readids1");

}

exit;

**Script 2 “deplexstep2.pl” :**

#!/usr/bin/perl

unless ($#ARGV == 0)

{

print "Usage: deplexstep2.pl S5_P1SampleList2.txt";

die;

}

open (INLIST, "<$ARGV[0]") || die;

# replace 'XXX' with your input and output directories

$indir = " XXX ";

$outdir = “XXX ";

# Loops through the list of your samples (' S5_P1SampleList2.txt ') and performs the commands for each one

while (<INLIST>) {

$lib = $_;

chomp($lib);

# A shortcut to read or write a file for each of your samples, each file having the same extension

$fa1 = $lib . ".fasta";

$readidsa = $lib . "a_ids.txt";

$readidsb = $lib . "b_ids.txt";

$readids2 = $lib . "_ab_ids.txt";

# combine the list of sequence names for 'a' and 'b' matches

system("cat $outdir/$readidsa $outdir/$readidsb >> $outdir/$readids2");

# split the trimmed fasta file into reads specific to each sample. Replace 'XX' with the name of your trimmed fasta file (output from mothur)

my $command1 = 'perl -ne'."'".'if(/^>(\S+)/){$c=$i{$1}}$c?print:chomp;$i{$_}=1 if'." @ARGV'"." $outdir/$readids2 $indir/XX.fasta > $outdir/$fa1";

system ($command1);

}

exit;

**Mitigating tag-jumping**

In this supplementary section we outlined the steps taken to mitigate the combined effects of tag-jumping (after Dunn *et al.* 2018). As detailed in the methods section of the manuscript, using Usearch v9.2.64 we initially removed those sequences that appeared fewer than 10 times in a sample. The bioinformatics pipeline was then run to the end, giving a preliminary output table on which decisions over cut off levels were made. Since only samples testing positive for a PCR product after gel electrophoresis were pooled, samples yielding no PCR band should not occur in the dataset. We therefore screened the preliminary output table for occurrence of any samples positive for Aphid DNA that were not pooled. The highest sum of reads found in such samples was then applied as a new threshold and the bioinformatics pipeline was re-run from the Usearch step onwards, using the newly identified thresholds in the minuniquesize command. Thus, all samples including less than this number of reads were excluded from any further analysis. For pool 1 the read threshold was set to 12 giving 8.06 % of false positives and in pool 2 to 96 giving 7.00% false positives (false positive percentages are percentage of unpooled samples showing positive for aphid DNA).

Reference:

Dunn JC, Stockdale JE, Moorhouse‐Gann RJ, McCubbin A, Hipperson H, Morris AJ, et al. The decline of the Turtle Dove: Dietary associations with body condition and competition with other columbids analysed using high‐throughput sequencing. Mol Ecol. 2018;27(16):3386–407.
